# Acceptability of Personal Sensing Among People With Alcohol Use Disorder: Observational Study

**DOI:** 10.2196/41833

**Published:** 2023-08-28

**Authors:** Kendra Wyant, Hannah Moshontz, Stephanie B Ward, Gaylen E Fronk, John J Curtin

**Affiliations:** 1 Department of Psychology University of Wisconsin-Madison Madison, WI United States

**Keywords:** personal sensing, digital therapeutics, mobile health, smartphone, alcohol use disorder, self-report, alcohol use, symptom monitoring, mental health, acceptability, alcohol intake, mobile phone

## Abstract

**Background:**

Personal sensing may improve digital therapeutics for mental health care by facilitating early screening, symptom monitoring, risk prediction, and personalized adaptive interventions. However, further development and the use of personal sensing requires a better understanding of its acceptability to people targeted for these applications.

**Objective:**

We aimed to assess the acceptability of active and passive personal sensing methods in a sample of people with moderate to severe alcohol use disorder using both behavioral and self-report measures. This sample was recruited as part of a larger grant-funded project to develop a machine learning algorithm to predict lapses.

**Methods:**

Participants (N=154; n=77, 50% female; mean age 41, SD 11.9 years; n=134, 87% White and n=150, 97% non-Hispanic) in early recovery (1-8 weeks of abstinence) were recruited to participate in a 3-month longitudinal study. Participants were modestly compensated for engaging with active (eg, ecological momentary assessment [EMA], audio check-in, and sleep quality) and passive (eg, geolocation, cellular communication logs, and SMS text message content) sensing methods that were selected to tap into constructs from the Relapse Prevention model by Marlatt. We assessed 3 behavioral indicators of acceptability: participants’ choices about their participation in the study at various stages in the procedure, their choice to opt in to provide data for each sensing method, and their adherence to a subset of the active methods (EMA and audio check-in). We also assessed 3 self-report measures of acceptability (interference, dislike, and willingness to use for 1 year) for each method.

**Results:**

Of the 192 eligible individuals screened, 191 consented to personal sensing. Most of these individuals (169/191, 88.5%) also returned 1 week later to formally enroll, and 154 participated through the first month follow-up visit. All participants in our analysis sample opted in to provide data for EMA, sleep quality, geolocation, and cellular communication logs. Out of 154 participants, 1 (0.6%) did not provide SMS text message content and 3 (1.9%) did not provide any audio check-ins. The average adherence rate for the 4 times daily EMA was .80. The adherence rate for the daily audio check-in was .54. Aggregate participant ratings indicated that all personal sensing methods were significantly more acceptable (all *P*<.001) compared with neutral across subjective measures of interference, dislike, and willingness to use for 1 year. Participants did not significantly differ in their dislike of active methods compared with passive methods (*P*=.23). However, participants reported a higher willingness to use passive (vs active) methods for 1 year (*P*=.04).

**Conclusions:**

These results suggest that active and passive sensing methods are acceptable for people with alcohol use disorder over a longer period than has previously been assessed. Important individual differences were observed across people and methods, indicating opportunities for future improvement.

## Introduction

### Personal Sensing

The World Health Organization’s Global Observatory for eHealth has concluded that “the use of mobile and wireless technologies to support the achievement of health objectives has the potential to transform the face of health service delivery across the globe” [1]. This conclusion applies to research and care for mental health as well as other traditional health services. These opportunities are now possible, in part, because of rapid advances in smartphones and related mobile technologies [2] and high levels of smartphone access across race, socioeconomic status, geographic region, and other demographic characteristics [3].

Personal sensing may become an important component of these digital health advances [4]. Personal sensing is a method for longitudinal measurement in situ, that is, real-world measurement embedded in individuals’ day-to-day lives [5-7]. Raw data streams are collected using smartphones, wearable sensors, or other smart devices. These raw data streams can consist of self-reports or more novel data streams, such as geolocation, cellular communication, social media activity, or physiology. Subsequent processing can extract psychiatric or health-relevant measures of thoughts, feelings, behavior, and even interpersonal interactions.

Ecological momentary assessment (EMA), a personal sensing method that collects brief self-reports about momentary states multiple times per day, has been used for many years in short-term longitudinal studies of psychiatric disorders. For example, EMA research on substance use disorders has identified proximal causes and risk factors for drug craving and relapse [8-10]. It has also characterized the time course and nature of drug withdrawal [11,12]. Much of this research could not have been accomplished with other measurement methods.

More recently, research using personal sensing of raw data streams other than self-reporting is emerging for mental health, including alcohol and other substance use disorders. This includes methods to sense geolocation [13-16], cellular communication [14-16], sleep [17], and physiology [15,16,18], for example. These alternative personal sensing methods provide benefits and opportunities that are not possible with EMA. For example, many of these data streams can be sensed passively such that they have a very low assessment burden. This may allow their use for long-term longitudinal monitoring of participants that would not be feasible with EMA, which requires more active effort for data collection.

Personal sensing is a powerful tool in mental health research [19]. These data are inherently longitudinal, which allows observation of the temporal ordering of putative etiological mechanisms and their effects. Longitudinal measurement is also critical for many mental health constructs that display meaningful and often frequent temporal variation in a person (eg, psychiatric symptoms). Measures based on personal sensing data generally have high ecological validity because they are collected in situ. Personal sensing measures also have low retrospective bias because they are often collected in real time. Furthermore, personal sensing can derive measures from raw data streams (eg, in situ behavior, physiology, and interpersonal interactions) that are difficult or even impossible to obtain through other traditional research measurement methods.

Personal sensing may have even higher value in the future for mental health clinical applications that target patient mental health care than it does for research [7,20,21]. Data collected by personal sensing methods may be used for preliminary screening of psychiatric disorders [22,23]. These methods can also be used to monitor psychiatric symptoms or even predict the future risk of symptom recurrence or other harmful behaviors (eg, suicide attempts and risky or otherwise harmful drinking episodes) [24-27]. For alcohol and other substance use disorders, there is emerging research on using sensed data to predict craving [13,18]; alcohol [15,27-29], cannabis [16], or opioid use [14]; and lapses or relapse [14,30,31]. Personal sensing measures or risk indicators may be shared, with patient consent, to health care providers to allow for cost-effective, targeted allocation of limited mental health resources to patients with the greatest or most urgent need [32]. Personal sensing has the potential to support precision mental health care by adapting and timing interventions based on characteristics of the patient and the moment in time [33-35]. These applications of personal sensing are currently aspirational rather than available for clinical implementation. However, clinical research is advancing rapidly toward these goals [14,30,36].

Mental health research and applications with emerging, often more passively sensed, novel data streams such as geolocation and cellular communication are still nascent. This research has predominantly involved *proof-of-concept* studies that typically include only healthy controls or other convenience samples rather than people with psychiatric disorders [16,17]. It has also often used very small sample sizes or short monitoring periods [15,16,18]. Recent reviews of this emerging literature have highlighted gaps in reporting on participant exclusions, attrition, and adherence that are necessary to assess selection biases and the feasibility of these novel personal sensing methods [37-39].

### Acceptability of Personal Sensing

Further development and use of personal sensing necessitates a better understanding of its acceptability to research participants and patients targeted for mental health applications. Will individuals consent to the use of personal sensing methods? Will they opt in to allow passive measurement methods? Can they sustain the behaviors necessary for active measurement methods for longer periods? Do they perceive specific personal sensing methods to be burdensome or dislike them? Answers to these questions about the acceptability of personal sensing methods are central to their feasibility for both mental health research and applications.

The acceptability of a personal sensing method may be influenced by the degree of active effort required from the participant or patient to collect the raw data (ie, the method’s assessment burden) and other factors (eg, sensitivity of the data collected). As such, acceptability may vary across different personal sensing methods, and comparisons across methods within the same individuals are thus warranted. Furthermore, a comprehensive assessment of both behavioral measures (eg, adherence) and subjective perceptions of acceptability may better anticipate potential issues for recruitment, consent, adherence, and attrition when they are used for either research or clinical applications.

Much of what is known about the acceptability of personal sensing is limited to EMA. Studies that have assessed participants’ perceptions of EMA methods have generally concluded that they are acceptable to participants from both nonclinical and clinical samples [40-44]. Similarly, participants displayed moderate or better adherence with respect to response rates, even with a relatively high sampling density (eg, 6-9 daily assessments) [40,45,46]. However, these studies generally assessed participants’ perceptions and adherence over short monitoring periods (ie, 2-6 weeks). Less is known about the use of EMA over longer monitoring periods (eg, months), as would be necessary for clinical applications.

Existing research also raises some concern about perceptions and adherence to EMA protocols in patients with alcohol and other substance use disorders relative to other groups. Specifically, a recent meta-analysis confirmed decreased adherence to EMA protocols in patients with substance use disorder diagnoses versus recreational substance users [47]. Furthermore, another meta-analysis [48] concluded that adherence rates did not differ between healthy and psychiatric samples, more generally. These meta-analyses combined suggest that adherence concerns may be limited to applications with patients with alcohol and other substance use disorders rather than all psychiatric disorders. For these reasons, it is important to further study the acceptability of EMA in samples with alcohol and other substance use disorders.

Far less is known about participants’ perceptions and adherence to passive personal sensing methods. Some research has presented hypothetical scenarios to either community or psychiatric samples to assess their perceptions about personal sensing methods [49-51]. Participants’ willingness to share sensed data appears to vary according to the data type (eg, sleep, geolocation, and social media activity). However, it is difficult to determine how well participants’ perceptions in these hypothetical scenarios would generalize to the real-world collection of these data. In addition, it is impossible to measure attrition and adherence outside the explicit implementation of these sensing methods.

Preliminary research has begun to examine perceptions and adherence during real-world use of passive personal sensing methods. However, this research has generally been limited by small sample sizes [52,53]; the use of convenience samples (eg, students and community participants) [41,52,54]; short monitoring duration [52,53,55,56]; and coarse, incomplete, or aggregate reporting of perceptions, adherence, and related participant behaviors [41,52,53]. These are important initial efforts, but more research into the feasibility of personal sensing methods is clearly warranted.

### Study Goals

This study reports on the acceptability of both active and passive personal sensing methods in a sample of participants with moderate to severe alcohol use disorder (AUD). These participants were enrolled early in their recovery period (ie, 1-8 weeks after becoming abstinent) and followed for 3 months. We used active personal sensing methods to collect EMA, daily audio check-ins, sleep quality, and selected physiology. We primarily used passive methods to collect geolocation, cellular communication logs, and SMS text message content. We assessed the participants’ choices regarding their participation in the study at various stages of the study procedure (eg, consent, enrollment, and data collection), their choice to opt in to provide data associated with each personal sensing method, and their reasons for discontinuation when available. For active measures, we also assessed their adherence for providing raw data streams for up to 3 months of their study participation. Finally, we assessed participants’ subjective perceptions of the acceptability of each of these personal sensing methods separately by self-report. We believe that these data provide insight into the feasibility of using numerous personal sensing methods with individuals with AUD, a highly stigmatized psychiatric disorder.

## Methods

### Research Transparency

We value the principles of research transparency that are essential for the robustness and reproducibility of science [57]. Consequently, we maximized transparency using several complementary methods. First, we reported how we determined our sample size, all data exclusions, all manipulations, and all available measures in the study [58]. Second, we completed a transparency checklist, which can be found in Multimedia Appendix 1 [59]. Third, we made the data, analysis scripts and annotated results, self-report surveys, and other study materials (eg, consent form and recruitment flyer) associated with this report publicly available through a study page on the Open Science Framework [60].

### Participants

#### Parent Project for Study Data

This study provides analyses to address the first aim of a larger grant-funded parent project (R01 AA024391) [61]. The broad goal of the project has been to develop a temporally precise machine learning algorithm to predict future lapses back to alcohol use in the next week, the next day, and the next hour. This algorithm will be integrated within an innovative digital therapeutic to support recovery for patients with alcohol and other substance use disorders—The Addiction Comprehensive Health Enhancement Support System (Center for Health Enhancement Systems Studies) [30,62,63]. This algorithm can be used to support patients to engage in ongoing self-monitoring of their recovery and to select, time, and adapt digital interventions to meet patients’ momentary needs during their recovery. We selected sensing methods that we believed would be well positioned to collect raw data streams to allow us to engineer machine learning features (ie, predictors) that tap into key constructs from the Relapse Prevention model [64-67], such as craving, affect, stressors, lifestyle imbalances, high-risk situations, self-efficacy and confidence, and abstinence violation effects. We focused on both active (eg, EMA) and passive (eg, geolocation and cellular communication logs) sensing methods to allow us to balance the potential predictive power and assessment burden. We sensed many of these raw data streams at high sampling rates to allow for temporally precise prediction (ie, up to 1-hour resolution) of lapse risk that may be necessary to deliver *just-in-time* digital interventions [33,68,69].

As a first step toward this broad goal of developing a lapse risk prediction algorithm, this study examined issues related to acceptability and feasibility (aim 1 of the grant) of collecting these actively and passively sensed raw data streams from individuals in early recovery from an AUD. We used all the available participants from the parent project for this study, and the sample size was determined based on power analyses for the aims of the project. We collected study data between 2017 and 2019.

#### Ethics Approval

All procedures were approved by the University of Wisconsin-Madison Institutional Review Board (Study #2015-0780).

#### Recruitment, Exclusion, and Inclusion Criteria

We recruited participants in early recovery (1-8 weeks of abstinence) from AUD in Madison, Wisconsin, United States, to participate in a 3-month longitudinal study. Participants were recruited through print and targeted digital advertisements and partnerships with treatment centers.

We excluded participants if they exhibited severe symptoms of psychosis or paranoia (defined as scores >2.2 or 2.8, respectively, on the psychosis or paranoia scales of the Symptom Checklist–90 [70]).

To be included, we required that participants (1) were aged ≥18 years; (2) were able to write and read in English; (3) had at least moderate AUD (≥4 Diagnostic and Statistical Manual of Mental Disorders, Fifth Edition symptoms); (4) were abstinent from alcohol for at least 1 week, but no longer than 2 months; and (5) were willing to use a single smartphone (their personal phone or one provided by us) while enrolled in the study.

**Figure 1:**
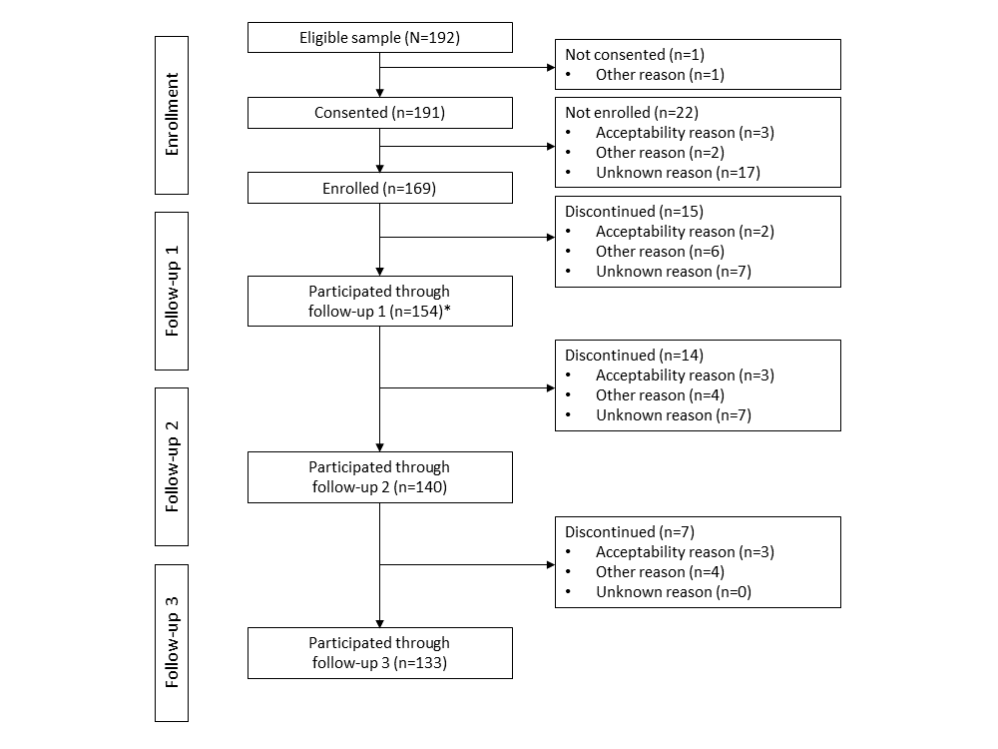
A flowchart of participant retention over the course of the 3-month study. This figure displays retention and attrition of all eligible participants at various stages from consent through study completion. It also displays the reasons for attrition categorized as because of acceptability, other reasons, or unknown. *Data of all participants who completed follow-up 1 were used in the analyses.

We assessed the inclusion and exclusion criteria using a brief phone screen followed by a more detailed in-person screening visit. A total of 192 participants were eligible for enrollment. Of these participants, 191 consented to participate in the study at the screening visit, and 169 subsequently enrolled in the study at the enrollment visit, which occurred approximately 1 week later. A total of 15 participants discontinued the study before their first monthly follow-up visit. The remaining 154 participants provided study measures for 1 (n=14), 2 (n=7), or 3 (n=133) months. A study participation flowchart is presented in Figure 1.

#### Compensation

We paid participants US $20 per hour for all time spent in the laboratory (ie, during screening, intake, and follow-up visits). In addition, we paid participants a US $99 bonus if they completed the study for the full 3-month duration. We also paid participants US $66 per month to offset the costs associated with their cellular plan and provided them with a smartphone for the study duration if they did not own one. Similarly, we provided them with bus transportation to and from the laboratory if needed.

For each sensing method, we paid participants bonuses (ranging from US $10 to US $25) if they had ≤10% missing data for that method each month. Specifically, if participants met these individual missing data thresholds, we paid them US $25 per month for EMA, US $25 per month for audio check-ins, US $15 per month for sleep quality data, US $15 per month for cellular communication logs and SMS text message content, and US $10 per month for geolocation. More details about these raw data streams are provided in the *Personal Sensing* section.

### Procedure

Participants completed 5 study visits over the course of approximately 3 months. Participants first attended a screening visit where we determined eligibility, obtained informed consent, and collected self-report measures of individual differences (eg, demographics and drug and alcohol use history). We scheduled eligible and consented participants to enroll in the study approximately 1 week later. During this enrollment visit, we collected additional self-report and interview measures. Participants completed 3 additional follow-up visits that occurred about every 30 days. We collected self-report and interview measures and downloaded cellular communication logs (ie, SMS text messages and phone calls) during these visits. Finally, we collected various raw data streams (eg, geolocation, and EMA) using personal sensing to monitor the participants throughout the 3-month study period. We informed the participants that we were collecting these data to develop an algorithm that could be used in the future to monitor for relapse risk. We did not provide them any further information about how each sensed data stream might be used in this algorithm. They were also not provided with any feedback or clinical interventions based on the sensing data that were collected from them. Furthermore, there were no consequences for continued study participation if participants lapsed back to alcohol use during the study. However, for human subjects reasons, we did offer brief motivational interviewing interventions to participants if they reported any alcohol use to the study staff. Participants were not required to participate in these interventions, but we offered it to them as support to maintain their recovery, if desired. Additional information about all these procedures (eg, recruitment flyer, consent form, and all surveys) can be found on the study’s Open Science Framework page [60].

### Personal Sensing

#### Overview

Personal sensing methods can be coarsely classified as active or passive. Active personal sensing requires active effort from the participant to provide the raw data streams, whereas passive personal sensing data are collected automatically (either asynchronously or continuously) with little to no effort required by the participant. Our study obtained several active signals that varied to a certain degree in the amount of effort required by the participants. Specifically, we used active methods to collect EMA, daily audio check-ins, sleep quality, and selected physiology. We primarily used passive methods to collect geolocation, cellular communication logs, and SMS text message content. More information about data collection and related procedures for each raw data stream is provided in the following sections.

#### EMA

Participants completed a brief EMA (7-10 questions) 4 times each day following reminders from us that were sent by SMS text message. These SMS text messages included a link to a Qualtrics (Qualtrics XM) survey that was optimized for completion on their smartphone. All 4 EMAs included items that asked about any alcohol use that had not yet been reported, current affective state (pleasantness and arousal), greatest urge to drink alcohol since the last EMA, any pleasant or positive events and any hassles or stressful events that occurred since the last EMA, and any exposure to risky situations (ie, people, places, or things) since the last EMA. The first EMA each day asked an additional 3 questions about how likely participants were to encounter a risky situation, to encounter a stressful event, and to drink alcohol in the upcoming week. The first and last EMAs of the day were scheduled within 1 hour of the participants’ typical wake and sleep times. The other 2 EMAs were each scheduled randomly within the first and second halves of the participants’ typical day. All the EMAs were separated from each other by at least 1 hour. Participants were required to agree to complete the EMAs for the duration of the study to participate in the study.

#### Audio Check-In

Participants recorded a diary-style audio response on their smartphone to an open-ended prompt each day, following a reminder from us that was sent via SMS text message. They responded to the prompt, “How are you feeling about your recovery today?” which stayed the same throughout the entire study. We instructed them that their responses should be approximately 15 to 30 seconds in duration. These recordings were sent to us via SMS text message. Participants were not required to complete audio check-ins to participate in the study, but the associated monthly sensing method compensation bonus was not provided unless they met the missing data thresholds each month (≤10% missing).

#### Geolocation

We continuously collected participants’ moment-by-moment geolocation data using location services on their smartphones in combination with a commercial app that accessed these geolocation data and saved them in the cloud. Participants were not required to provide these data to participate in the study, but the associated monthly sensing method compensation bonus was not available if they did not provide these data each month. Participants opted in at the start of the study to provide these data by installing the app on their phone. They were allowed to opt out at any later point by simply uninstalling the app. At the start of the study, we used the Moves app (ProtoGeo Oy). However, Facebook acquired ProtoGeo Oy and shut down use of the Moves app in July 2018. At this point, we switched to using the FollowMee (FollowMee LLC) GPS tracking mobile app. Measurement of geolocation required only the initial installation of the app by the participants. Subsequent measurement and transfer of the data to the cloud was completed automatically with no input or effort by the participant. Both apps allowed participants to temporarily disable location sharing if they deemed it necessary for short periods.

#### Cellular Communication Logs

We collected cellular communication logs that included metadata about smartphone communications involving both SMS text messages and phone calls. For each communication entry, these logs include the phone number of the other party, the type of call or message (ie, incoming, outgoing, missed, or rejected), the name of the party if listed in the phone contacts, the date and time the message or call occurred, whether the log entry was read (SMS text messages only), and the duration of the call (voice calls only). These data are saved passively on the phone with no additional input or effort from the participant. We downloaded these logs from participants’ phones at each monthly follow-up visit. Participants were not required to provide these data to participate in the study, but the associated monthly sensing method compensation bonus was not available if they did not provide these data each month. Participants opted in to provide these data when they allowed us to download their data at the study visit. Participants were informed that they could delete any SMS text message or voice call log entries before the download, if they desired.

#### SMS Text Message Content

We also collected the message content from the participants’ SMS text messages on their smartphones. As with the logs, content from individual SMS text messages is saved passively on the phone with no additional input or effort from the participant. We downloaded SMS text message content (bundled with the cellular communication logs in the same files) at each monthly follow-up visit, and participants could delete SMS text messages before the download. We did not have a parallel method to gain access to phone call content. Thus, we had metadata from cellular communication logs for both SMS text messages and phone calls but had the content of the communication only for SMS text messages.

#### Sleep Quality

We collected information about participants’ sleep duration, timing, and overall quality with a Beddit Sleep Monitor (Beddit Oy Inc) that was placed in their beds and connected to their smartphones. We used an early version of the sleep monitor that required participants to actively start and stop the monitor when they entered and exited their beds each night and morning, respectively. These data are available for only 87 participants because Beddit Oy was acquired by Apple Inc during the data collection for this study. Apple discontinued cloud support for data collection with the sleep monitor in November 2018, which prevented its further use for our remaining participants. Participants were not required to provide these data to participate in the study, but the associated monthly sensing method compensation bonus was not available if they did not provide these data each month. Participants opted in at the start of the study to provide these data by installing the app on their phone. They were allowed to opt out at any later point by simply uninstalling the app.

#### Physiology

We continuously monitored participants’ physiology (heart rate, electrodermal activity, and skin temperature) using an early version of the Empatica E4 (Empatica Inc) wristband monitor. However, this early version did not adequately support the Bluetooth streaming of data to the cloud. Instead, participants had to manually connect the wristband each night to a tablet we provided to upload their data. This limitation and other software bugs made the use of the wristband too complicated for many participants. Therefore, we discontinued the use of the wristband after we collected data from 9 participants. Given the small sample size, we did not include the wrist band data in our primary analyses. We provide self-reported acceptability ratings for this signal from this small sample in Figure S1 in Multimedia Appendix 2.

### Measures

#### Individual Differences

We collected self-report information about demographics (age, sex, race, ethnicity, education, employment, personal income, and marital status) and drug and alcohol use history (AUD milestones; number of quit attempts; lifetime history of treatment for AUD; lifetime receipt of medication for AUD; Diagnostic and Statistical Manual of Mental Disorders, Fifth Edition AUD symptom count; lifetime drug use; and current drug use) at the screening visit.

#### Behavioral Measures of Acceptability

A coarse assessment of the acceptability of personal sensing methods can be made based on the participants’ behaviors. Specifically, we assessed 3 categories of behavior. First, we assessed participants’ choices regarding their participation in the study at various stages of the study procedure (eg, consent, enrollment, and data collection) and their reasons for discontinuation when available. Second, we assessed their choice to opt in to provide data associated with each personal sensing method. Participants were allowed to participate in the study without opting in for any specific personal sensing method other than EMA. Finally, for a subset of the active measures (EMA and audio check-in), we assessed their behavioral adherence for up to 3 months of study participation.

#### Self-Reported Measures of Acceptability

To assess participants’ subjective experience of the acceptability of the personal sensing methods in this study, each month, they rated each method on 3 acceptability-relevant dimensions (Multimedia Appendix 3). Specifically, participants were asked to indicate how much they agree or disagree with each statement on a 5-point bipolar scale (strongly disagree, disagree, undecided, agree, or strongly agree) for personal sensing signals: (1) “[Personal sensing method name] interfered with my daily activities,” (2) “I disliked [Personal sensing method name],” and (3) “I would be willing to use [Personal sensing method name] for 1 year to help with my recovery.”

The interference item (item 1) was collected only for the active methods because the passive methods require no effort and therefore cannot interfere with daily activities. Dislike and willingness to use for 1 year (items 2 and 3, respectively) were collected for all methods.

#### Participant Feedback

We also solicited open-ended feedback about the participants’ experiences with each personal sensing method. Each month, participants were prompted as follows: “Tell us your general thoughts, whether positive or negative, about your experience completing [Personal sensing method name].” These qualitative data provided another method through which to assess participants’ perceptions of the acceptability of these methods.

### Data Analytic Strategy

We conducted all analyses in R version 4.1.1 (R Core Team) [71] using RStudio [72] and the *tidyverse* ecosystem of packages [73].

#### Behavioral Measures of Acceptability

We provide descriptive data on participants’ choices about their participation in the study at various stages of the study procedure (eg, consent, enrollment, and data collection). We provide both coarse and more granular tabulations of their reasons for discontinuation when available. We report the percentages of participants who opted in to provide us with the raw data streams we collected via personal sensing. We also report adherence for 2 active personal sensing methods (EMA and audio check-in). Formal measures of adherence could not be calculated for geolocation, cellular communication logs, SMS text message content, and sleep quality because it was not possible to distinguish between low volumes of data owing to adherence (eg, deleting phone calls or messages, turning off location services on the phone, and failing to start sleep monitoring at bedtime) and other valid reasons (no calls made during the day, no movement, and erratic sleep patterns).

#### Self-Reported Measures of Acceptability

Participants responded to the 3 self-report items related to acceptability (interference, dislike, and willingness to use for 1 year) on a 5-point bipolar scale (strongly disagree, disagree, undecided, agree, or strongly agree). We retained these ordinal labels for visual display of these data in figures but ordered the labels such that higher scores represented greater acceptability (ie, strongly agree for willingness to use for 1 year and strongly disagree for interference and dislike). For the analyses, we recoded these items to a numeric scale ranging from −2 to 2, with 0 representing the neutral (undecided) midpoint and higher scores representing greater acceptability.

Participants responded to these items at each monthly follow-up visit. Therefore, participants had up to 3 responses for each item, depending on when they ended their participation. We analyzed their last available response in our primary analyses to allow us to include all participants and to represent their final perception of each personal sensing signal. However, mean responses across each time point remained relatively constant for all signals (Figure S2 in Multimedia Appendix 2).

To detect the mean perceptions of the personal sensing signals that diverge from neutral (ie, mean responses to any items that are different from 0 or undecided), we conducted 2-tailed, 1 sample *t* tests for the 3 self-report items for each personal sensing signal. To examine relative perceptions of the signals, we compared perceptions of the active versus passive categories of signals using 2-tailed, within-sample *t* tests. Participants did not provide ratings of interference for passive signals so the comparisons of active versus passive categories were limited to dislike and willingness to use for 1 year. Due to the high proportion of missing data for sleep quality, we excluded this signal from these analyses and the intraclass correlations described next. Comparisons among all personal sensing signals using 2-tailed, within-sample *t* tests for each of the 3 self-report items are reported in Table S1 in Multimedia Appendix 2.

Finally, we conducted 2 analyses to examine the consistency of perceptions across personal sensing signals (eg, Do participants who dislike 1 signal also dislike the other signals?). First, we calculated bivariate correlations among the personal sensing signals for each item. Second, we calculated intraclass correlations (single, case 3 [74]) separately for each item to quantify agreement in participants’ perceptions across the signals.

#### Participant Feedback

We have provided all raw participant responses, organized by the sensing method, in Tables S2 to S6 in Multimedia Appendix 2. In addition, we have provided representative positive and negative evaluations organized by guiding themes (acceptability, sustainability, benefits, trust, and usability) developed from our literature review in Table S7 in Multimedia Appendix 2.

## Results

### Participant Characteristics

A total of 154 participants completed at least 1 monthly follow-up visit and provided self-reported acceptability ratings for interference, dislike, and willingness to use for 1 year. These participants served as the primary sample for our analyses. Participants were mostly White (134/154, 87%) and non-Hispanic (150/154, 97.4%). Half (77/154, 50%) of our research participants were female, and the mean age was 41 (SD 11.9) years. Table 1 presents detailed demographic information. Table 2 presents the information relevant to lifetime drug and alcohol use for these participants. We compared demographics and drug and alcohol use information for participants who were included in the analyses with those of eligible participants who did not provide study measures (ie, did not enroll or discontinued before the first month follow-up; n=36) and found no significant differences (Table S8 in Multimedia Appendix 2 presents details on these analyses).

**Table 1:** Participant demographic data (N=154).

Characteristics		Participants
Age (years), mean (SD)		41 (11.9)
**Sex, n (%)**		
	Female	77 (50)
	Male	77 (50)
**Race, n (%)**		
	American Indian or Alaska native	3 (1.9)
	Asian	2 (1.3)
	Black or African American	8 (5.2)
	White	134 (87)
	Other or multiracial	7 (4.5)
**Hispanic, Latino, or Spanish origin, n (%)**		
	Yes	4 (2.6)
	No	150 (97.4)
**Education, n (%)**		
	Less than high school or GED^a^ degree	1 (0.6)
	High school or GED	15 (9.7)
	Some college	43 (27.9)
	2-Year degree	14 (9.1)
	College degree	58 (37.7)
	Advanced degree	23 (14.9)
**Employment, n (%)**		
	Employed full time	72 (46.8)
	Employed part time	27 (17.5)
	Full-time student	7 (4.5)
	Homemaker	1 (0.6)
	Disabled	7 (4.5)
	Retired	8 (5.2)
	Unemployed	19 (12.3)
	Temporarily laid off, sick leave, or maternity leave	3 (1.9)
	Other, not otherwise specified	10 (6.5)
Personal income (US $), mean (SD)		34,233 (31,543)
**Marital status, n (%)**		
	Never married	69 (44.8)
	Married	33 (21.4)
	Divorced	45 (29.2)
	Separated	5 (3.2)
	Widowed	2 (1.3)

^a^GED: General Educational Development.

**Table 2:** Participant drug and alcohol use history data (N=154).

Characteristics			Participants
**Alcohol use disorder milestones, mean (SD)**			
	Age of first drink	14.6 (2.9)	
	Age of regular drinking	19.5 (6.5)	
	Age at which drinking became problematic	27.9 (9.6)	
	Age of first quit attempt	31.6 (10.4)	
	Number of quit attempts	9.1 (31.1)	
**Lifetime history of treatment (can choose more than 1), n (%)**			
	Long-term residential (>6 mo)	8 (5.2)	
	Short-term residential (<6 mo)	51 (33.1)	
	Outpatient	77 (50)	
	Individual counseling	100 (64.9)	
	Group counseling	65 (42.2)	
	Alcoholics anonymous or narcotics anonymous	96 (62.3)	
	Other	41 (26.6)	
**Received medication for alcohol use disorder, n (%)**			
	Yes	62 (40.3)	
	No	92 (59.7)	
DSM-5^a^ alcohol use disorder symptom count, mean (SD)			8.9 (1.9)
**Lifetime drug use, n (%)**			
	Tobacco products (eg, cigarettes, chewing tobacco, and cigars)	122 (79.2)	
	Cannabis (eg, marijuana, pot, grass, and hash)	131 (85.1)	
	Cocaine (eg, coke and crack)	86 (55.8)	
	Amphetamine type stimulants (eg, speed, diet pills, and ecstasy)	81 (52.6)	
	Inhalants (eg, nitrous, glue, petrol, and paint thinner)	36 (23.4)	
	Sedatives or sleeping pills (eg, Valium, Serepax, and Rohypnol)	72 (46.8)	
	Hallucinogens (eg, LSD^b^, acid, mushrooms, PCP^c^, and Special K)	88 (57.1)	
	Opioids (eg, heroin, morphine, methadone, and codeine)	65 (42.2)	
**Current drug use^d^, n (%)**			
	Tobacco products (eg, cigarettes, chewing tobacco, and cigars)	84 (54.5)	
	Cannabis (eg, marijuana, pot, grass, and hash)	52 (33.8)	
	Cocaine (eg, coke and crack)	4 (2.6)	
	Amphetamine type stimulants (eg, speed, diet pills, and ecstasy)	11 (7.1)	
	Sedatives or sleeping pills (eg, Valium, Serepax, and Rohypnol)	24 (15.6)	
	Hallucinogens (eg, LSD, acid, mushrooms, PCP, and Special K)	9 (5.8)	
	Opioids (eg, heroin, morphine, methadone, and codeine)	9 (5.8)	

^a^DSM-5: Diagnostic and Statistical Manual of Mental Disorders, Fifth Edition.

^b^LSD: lysergic acid diethylamide.

^c^PCP: phencyclidine.

^d^Current refers to the previous month’s drug use reported at follow-up visits 1 or 2.

### Behavioral Measures of Acceptability

#### Participation

Figure 1 shows participant attrition and discontinuation at each phase of the study. Of the 192 eligible participants at screening, only 1 did not consent to participate after hearing the details of the study. Enrollment occurred during a second visit 1 week later. A total of 169 participants completed enrollment.

In Figure 1, we coarsely tabulated reasons stated by participants for discontinuation as because of acceptability, other reasons, or unknown. In total, 11 (5.7%) of the 192 eligible participants were lost due to acceptability-relevant causes (eg, no longer interested, nonadherence to sensing methods, or citing study demands as too burdensome). Other reasons for discontinuation not related to the acceptability of the signals include circumstances such as moving or no longer wishing to abstain from alcohol. It is notable that 31 (16.1%) of the 192 participants were lost to follow-up, such that we had no information about their reasons for discontinuation. We provide a more granular tabulation of these reasons for discontinuation in Table S9 in Multimedia Appendix 2.

#### Opt-In and Adherence

All participants who completed follow-up 1 (154/154, 100%) opted in to provide data for EMA, sleep quality, and most of the passive personal sensing methods (geolocation and cellular communication logs) throughout their entire participation period. Out of 154 participants, 1 (0.64%) did not provide SMS text message content, and 3 (1.9%) did not provide any audio check-ins during the study.

**Figure 2:**
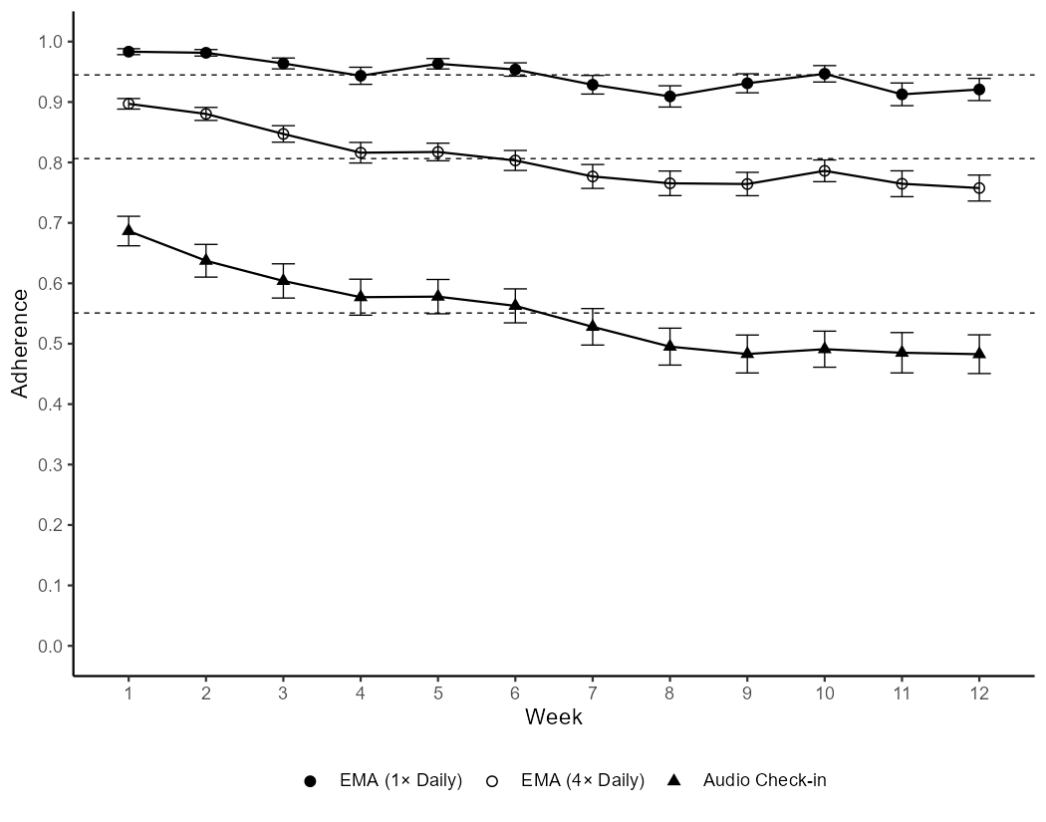
Adherence over time for EMA (once daily), EMA (4 times daily), and audio check-in. Plot depicts mean adherence rates for each week on study. Mean SE is depicted by the solid error bars. Overall mean adherence rate is depicted by the dashed line. The sample size was 154. EMA: ecological momentary assessment.

Daily adherence rates were relatively high for EMA, such that on 94.1% of the study days, participants completed at least 1 of the 4 EMAs. On average, participants completed 3.2 (SD 0.64) EMAs every day. The overall adherence rate for all requested EMAs was .80. The participants’ adherence rate for audio check-in was .54 (Figure S3 in Multimedia Appendix 2 contains more information on this distribution), that is, of their total days in the study, participants completed an audio check-in on approximately half of the days. Figure 2 shows the mean weekly adherence to each of these methods for each week in the study. In Multimedia Appendix 2, we also report adherence for participants who completed the 3-month study compared with those who dropped out before completion (Figure S4 in Multimedia Appendix 2).

### Self-Reported Acceptability

#### Interference

**Figure 3:**
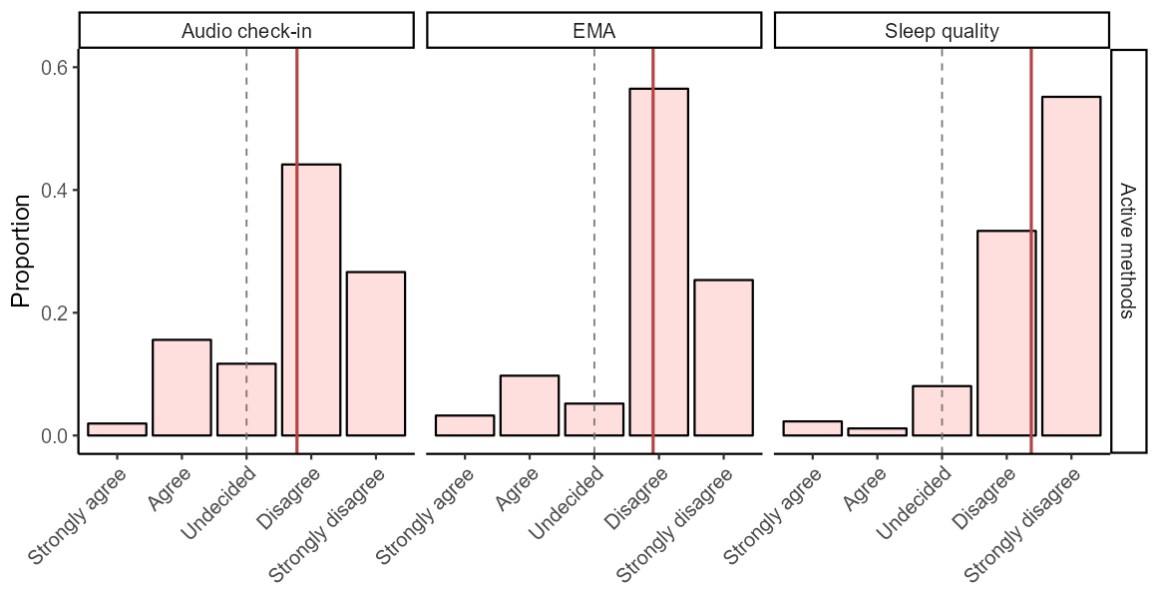
Ratings of interference by personal sensing data stream. Plot depicts mean responses to “[Personal sensing method name] interfered with my daily activities.” X axes are ordered to display a higher acceptability on the right side. For sleep quality, the sample size was 87; for all other data streams, the sample size was 154. The solid red lines represent the mean, and the dashed lines represent the neutral midpoint (undecided). All raw data streams had a significantly (*P*<.001) higher mean than the neutral midpoint. Interference ratings were collected only for active methods. EMA: ecological momentary assessment.

Figure 3 shows the distribution of the participants’ responses to the self-reported acceptability item about interference. Responses were grouped by personal sensing data stream and the amount of active effort required to collect it. Two-tailed, 1-sample *t* tests revealed that each mean interference score (depicted as the solid red line) was significantly (all *P*<.001) more acceptable than 0 (the gray dashed line indicating undecided). Table 3 reports the summary statistics for each 2-tailed, 1-sample *t* test and pairwise correlations between the personal sensing data streams. An intraclass correlation coefficient (ICC; type 3) showed that, on average, interference ratings were moderately consistent across the data streams (ICC=0.42, 95% CI 0.31-0.53).

**Table 3:** Bivariate and univariate statistics by acceptability and personal sensing data stream.

				1^a^		2		3		4		5		Value, n		Value, mean (SD)		*t* test (*df*)		Cohen *d*
**Interference**																				
	**Active methods**																			
		Audio check-in	1.00		0.43		−0.06		—^b^		—		154		0.78 (1.07)		9.05 (153)^c^		0.73	
		EMA^d^	0.43		1.00		0.24		—		—		154		0.91 (0.99)		11.37 (153)^c^		0.92	
		Sleep quality	−0.06		0.24		1.00		—		—		87		1.38 (0.87)		14.86 (86)^c^		1.59	
**Dislike**																				
	**Active methods**																			
		Audio check-in	1.00		0.57		0.25		0.31		0.22		154		0.51 (1.28)		4.91 (153)^c^		0.40	
		EMA	0.57		1.00		0.26		0.36		0.25		154		0.96 (0.92)		12.92 (153)^c^		1.04	
		Sleep quality	0.25		0.26		1.00		0.41		0.33		87		1.10 (1.09)		9.45 (86)^c^		1.01	
	**Passive methods**																			
		Geolocation	0.31		0.36		0.41		1.00		0.67		154		1.03 (0.94)		13.51 (153)^c^		1.09	
		Cellular communication logs	0.22		0.25		0.33		0.67		1.00		154		0.90 (0.97)		11.45 (153)^c^		0.92	
		SMS text message content	0.34		0.28		0.19		0.62		0.69		154		0.58 (1.18)		6.07 (153)^c^		0.49	
**Willingness to use for 1 year**																				
	**Active methods**																			
		Audio check-in	1.00		0.44		0.44		0.51		0.47		154		0.73 (1.28)		7.09 (153)^c^		0.57	
		EMA	0.44		1.00		0.41		0.48		0.47		154		0.64 (1.22)		6.47 (153)^c^		0.52	
		Sleep quality	0.44		0.41		1.00		0.53		0.47		87		0.85 (1.28)		6.19 (86)^c^		0.66	
	**Passive methods**																			
		Geolocation	0.51		0.48		0.53		1.00		0.65		154		0.94 (1.18)		9.83 (153)^c^		0.79	
		Cellular communication logs	0.47		0.47		0.47		0.65		1.00		154		0.84 (1.07)		9.76 (153)^c^		0.79	
		SMS text message content	0.47		0.39		0.39		0.60		0.84		154		0.74 (1.12)		8.21 (153)^c^		0.66	

^a^Initial columns (1-5) indicate bivariate correlations among data streams for each self-report acceptability measure. The final columns show the number of participants (n), mean and SD, *t* test statistic, and Cohen *d* effect size (*d*) for the 2-tailed, 1-sample *t* tests against 0 (undecided). Higher values indicate higher levels of acceptability.

^b^Not available.

^c^*P*<.001.

^d^EMA: ecological momentary assessment.

#### Dislike

**Figure 4:**
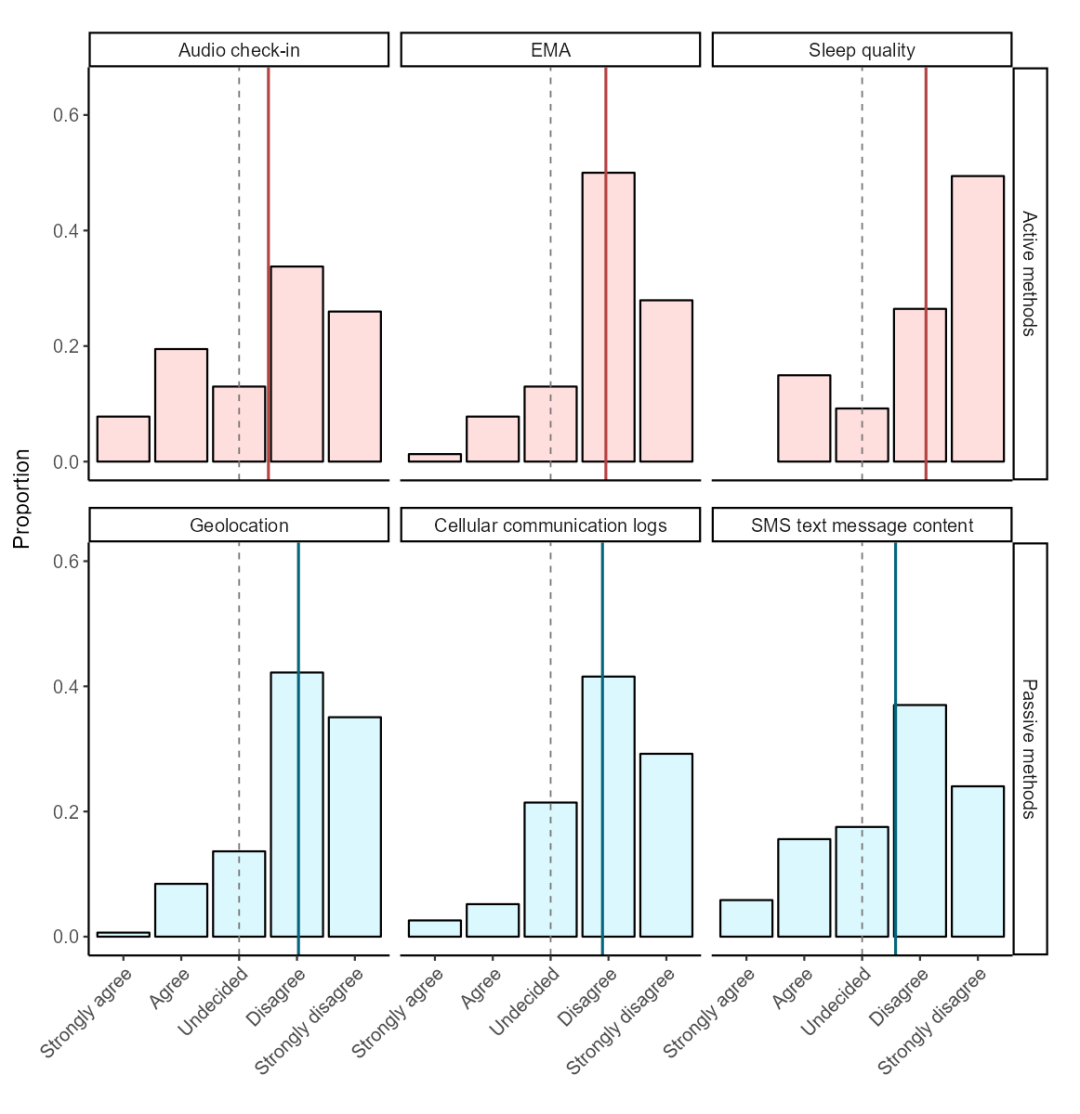
Ratings of dislike by personal sensing data stream. Plot depicts mean responses to “I disliked [personal sensing method name].” X axes are ordered to display a higher acceptability on the right side. For sleep quality, the sample size was 87; for all other data streams, the sample size was 154. The solid red and blue lines represent the mean, and the dashed lines represent the neutral midpoint (undecided). All raw data streams had a significantly (*P*<.001) higher mean than the neutral midpoint. Active methods are displayed in red, and passive methods are displayed in blue. EMA: ecological momentary assessment.

Figure 4 shows the distribution of participant responses to the self-reported acceptability item about dislike by the personal sensing data stream and the amount of active effort required to collect it. Two-tailed, 1-sample *t* tests revealed that each mean dislike score was significantly (all *P*<.001) more acceptable than 0. Table 3 reports the summary statistics for each 2-tailed, 1-sample *t* test and the pairwise correlations between the personal sensing data streams. An ICC (type 3) showed that, on average, the dislike ratings were moderately consistent across the data streams (ICC=0.42, 95% CI 0.35-0.48).

We also assessed the effect of active effort on the dislike ratings (see Figure S5 in Multimedia Appendix 2). We conducted a 2-tailed, paired-sample *t* test to compare the average dislike for active (eg, audio check-in and EMA) with passive (eg, geolocation, cellular communication logs, and SMS text message content) methods. Participants did not significantly differ in their dislike of active and passive methods (*t*_153_=1.21, *P*=.23; Cohen *d*=0.10).

#### Willingness to Use for 1 Year

**Figure 5:**
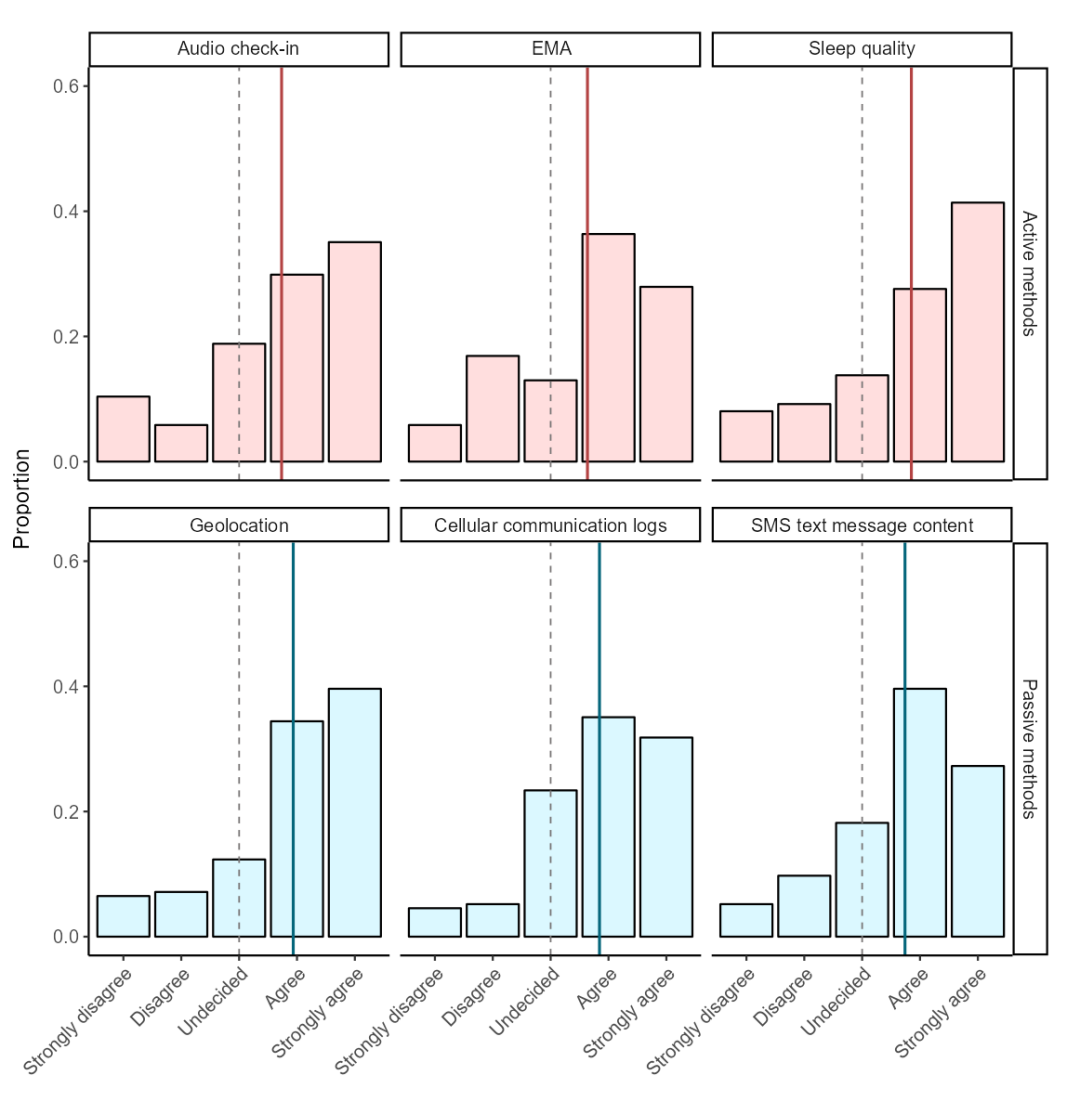
Ratings of willingness to use for 1 year by personal sensing data stream. Plot depicts mean responses to “I would be willing to use [Personal sensing method name] for 1 year to help with my recovery.” X axes are ordered to display a higher acceptability on the right side. For sleep monitoring, the sample size was 87; for all other data streams, the sample size was 154. The solid red and blue lines represent the mean, and the dashed lines represent the neutral midpoint (undecided). All raw data streams had a significantly (*P*<.001) higher mean than the neutral midpoint. Active methods are displayed in red, and passive methods are displayed in blue. EMA: ecological momentary assessment.

Figure 5 shows the distribution of participants’ responses to the self-reported acceptability item about willingness to use for 1 year for each personal sensing data stream (Figure S6 in Multimedia Appendix 2 contains additional information about willingness to use an EMA method once daily for 1 year). Two-tailed, 1-sample *t* tests revealed that each mean willingness score was significantly (all *P*<.001) more acceptable than 0. Table 3 reports the summary statistics for each 2-tailed, 1-sample *t* test and pairwise correlations between the personal sensing data streams. An ICC (type 3) showed that, on average, the willingness ratings were moderately consistent across the data streams (ICC=0.52, 95% CI 0.46-0.58).

We also assessed the effect of active effort on willingness ratings (see Figure S7 in Multimedia Appendix 2). We conducted a 2-tailed, paired-sample *t* test of the average willingness to use for 1 year for active (eg, audio check-in and EMA) and passive (eg, geolocation, cellular communication logs, and text message content) signals. Participants reported higher acceptability with respect to willingness for passive data streams (mean 0.80, SD 1) than active data streams (mean 0.70, SD 1.10; *t*_153_=2.12, *P*=.04; Cohen *d*=0.17).

#### Participant Feedback

In participants’ free-response feedback about each personal sensing data stream, we identified 5 themes: acceptability (“I had no issues with the daily EMA surveys. I felt that they kept me in check and were a reminder to not drink. I would not change it.”); sustainability (“I forgot I was being tracked, so it was not a big deal to me.”); benefits (“Was okay to have [geolocation tracking] done in the context of the study or for an app that would help me stay sober.”); trust (“I trusted the study group to not use my personal information for any other use.”); and usability (“I disliked saving my text messages. I like deleting them when I’m done.”). A representative sample of comments are provided for each theme in Table S7 in Multimedia Appendix 2. A full unedited list of participant comments for each personal sensing data stream has been presented in Tables S2 to S6 in Multimedia Appendix 2.

## Discussion

### Principal Findings

This study evaluated the acceptability of active and passive personal sensing methods for a variety of raw data streams and associated methods. To this end, we assessed participants’ choices and behaviors about both participating in the study and providing raw data streams for each method and their subjective perceptions of each sensing method. We focused on participants with moderate to severe AUD because they might have been expected to be less willing to share sensitive, private information owing to the stigma associated with their disorder [75]. However, if these sensing methods were acceptable to them, highly promising opportunities are now emerging to address their largely unmet treatment needs [76], with technological solutions that include digital therapeutics combined with personal sensing [77]. We have organized our discussion around 7 key conclusions from our analyses.

### Individuals With AUD Will Generally Accept the Use of Personal Sensing Methods

On the basis of our sample, it appears that individuals with AUD are indeed willing to provide these sensitive, personally sensed raw data streams based on their behavioral choices regarding consent, enrollment, and opt in for data collection in this study. All but one of the individuals (191/192, 99.5%) who were eligible to participate consented to the personal sensing procedures. Most of these individuals (169/191, 88%) also returned 1 week later to formally enroll in the study and begin to provide these data. Furthermore, all (169/169, 100%) of the participants who enrolled in the study explicitly opted in to provide the 3 arguably most sensitive passive data streams: geolocation, cellular communication logs, and SMS text message content.

These consent, enrollment, and opt-in numbers could be considered upper-bound and lower-bound estimates of the percentage of individuals who are willing to provide these raw data streams in a research setting. The very high percentage for consent may overestimate willingness because some of these individuals may have reconsidered their initial decision on further reflection such that they did not return for the next study visit to enroll formally. However, the still quite high enrollment percentage may underestimate the willingness to provide these data because some attrition was expected between consent and enrollment visits due to the instability associated with the early stages of recovery from AUD. In fact, Table S9 in Multimedia Appendix 2 indicates that almost half of the participants who consented but did not enroll may have done so for reasons other than their willingness to provide these raw data streams (eg, health concerns, no transportation to lab, and made repeated attempts to reschedule before discontinuing).

Participants’ explicit self-reports of their perceptions about the acceptability of these personal sensing methods were also generally consistent with their behavior. Specifically, on average, participants rated all the sensing methods as more favorable than the neutral midpoint (“undecided”) of the rating scales for all 3 dimensions we evaluated: interference, dislike, and willingness to use for 1 year. These self-report data combined with our behavioral measures suggest that all of these sensing methods can be considered for use with the majority of individuals with AUD.

Despite the aggregate positive perceptions of the full sample, nontrivial percentages of participants reported individual ratings that were more negative than the neutral midpoint across the sensing methods and specific self-report items. For example, 17.5% (27/154) of the participants agreed or strongly agreed that audio check-ins interfered with their daily activities. Approximately 25% of the participants agreed or strongly agreed that they disliked both the audio check-ins (42/154, 27.3%) and providing access to the content of their SMS text messages (33/154, 21.4%). Approximately 20% of the participants disagreed or strongly disagreed that they would be willing to use our sensing methods for audio check-ins (25/154, 16.2%), EMA (35/154, 22.7%), and SMS text message content (23/154, 14.9%) for 1 year to help with their recovery. This suggests that there is still a need to improve each of these sensing methods to make them more acceptable to a larger percentage of individuals. The free-response evaluations of each method provide a starting point to address participant concerns. However, our research participants did generally opt in and adhere to our sensing methods despite reporting these concerns. Therefore, the threshold at which these concerns will translate to barriers for use or adherence to these methods is unclear.

### Individuals Can Sustain the Use of Personal Sensing for Relatively Long Periods

Most enrolled participants were also able to sustain their commitment to providing these sensed data streams over time. More than 91% (154/169) provided at least 1 month of sensed data, and a large majority (133/169, 78.7%) provided data for all 3 months. As with enrollment statistics, these numbers also likely underestimate participants’ ability to sustain personal sensing because many of the participants who discontinued or did not complete the study reported reasons to stop their participation that were unrelated to personal sensing (eg, family crisis, relapse, and moved out of state). However, some participants (n=4) explicitly reported reasons that appeared related to personal sensing (eg, study demands were too burdensome). In addition, others who stopped participating may have been influenced by their experiences with personal sensing without formally reporting their concerns.

Participants who enrolled but then discontinued because of personal sensing methods may have been influenced more by issues related to the burden associated with active sensing rather than more general issues related to data sensitivity and privacy. Participants concerned about sharing passively sensed private information, such as their moment-by-moment location or cellular communication, would likely have had these concerns from the beginning, such that they would not have consented, enrolled, and then opted in to provide these sensitive data. However, the burden associated with active sensing (eg, 4 times daily EMA and daily audio check-ins) may not have been clear to them until they tried to sustain those methods over time. In our sample of participants, we saw evidence that many of them hardly thought about passively sensed data streams. On the other hand, some participants reported more discontent with actively sensed data streams as time progressed.

Existing research assessing the acceptability of sensing methods has been limited by short durations of monitoring, with very few studies extending beyond 6 weeks [53,55,56]. In addition, adherence has been shown to decrease after only a few weeks in some studies [43,48,78]. This study demonstrates that individuals can sustain their commitment to providing personally sensed data over time with limited drop-offs. These findings suggest that personal sensing methods may be viable in clinical settings where consistent, sustained monitoring would be necessary. Given this promise, future research should expand to longer durations to assess self-reported and behavioral acceptability beyond 3 months. Our group is exploring this directly by using personal sensing monitoring in individuals with opioid use disorder for a full year [14]. Methods that permit long-term monitoring are particularly important for clinical applications for individuals with substance use disorders, who require lifelong care that can adapt to their risk for relapse and corresponding recovery needs.

### Some Types of Active Personal Sensing Methods Are Generally Acceptable and Sustainable

The assessment burden may be expected to play a role in both the acceptability of active sensing methods and participant adherence to the associated procedures. Nonetheless, participants displayed relatively high adherence to the 4 times daily EMA (79.8% of EMAs completed on average). This is notable because our study duration of 3 months was substantially longer than typical studies using EMA, which often lasts only 2 to 4 weeks [47,48]. This increases confidence in the feasibility of this active sensing method for research and clinical applications that require longer monitoring periods. This level of adherence may be contingent on the measurement parameters used in our study (4 times daily survey of 7-10 items). In fact, even higher adherence may have been observed if the measurement was limited to 1 EMA per day, given that on average participants completed at least 1 of the 4 EMAs on 94.1% of the study days. Participants were also significantly more likely to report a willingness to use a once daily EMA compared with a 4 times daily EMA for 1 year. However, these findings should be interpreted cautiously. Participant self-reports to a once daily EMA method are not based on experience because they were expected to adhere to the 4 times daily EMA. From free-response comments, we saw that many of our participants had no issues with the 4 times daily EMA and some even enjoyed the frequent prompts. However, other participants suggested less-frequent prompts would be more practical.

Overall, there was some evidence that participants found passive sensing methods to be more acceptable than active sensing methods. Specifically, the mean ratings for willingness to use for 1 year were significantly higher for passive sensing methods than active sensing methods. However, the magnitude of this effect was small, and the mean willingness was significantly greater than the neutral midpoint for both the active and passive methods. In addition, there was no difference in the mean dislike ratings between the active and passive methods. Thus, the differences between the acceptability of the active and passive methods were small, inconsistent, and unlikely to be clinically meaningful. These comparisons between active and passive methods increase our confidence somewhat that the selective use of active measures, when necessary, may be acceptable to participants for relatively long periods. However, from this study, we cannot speculate strongly beyond 3 months.

Some sensing methods (eg, EMA and audio check-ins) will always require active input from users, but other methods may become more passive with further technological advances. For example, our sensing of sleep quality in this study used an early version of the Beddit Sleep Monitor that required participants to actively log when they entered and exited their bed during each period of sleep. However, later versions of Beddit automatically detect periods of sleep. Similarly, we discontinued the sensing of physiology with Empatica E4 in an early phase of our study because participants had to manually connect the wristband each night to a tablet to upload their data. This proved too burdensome and complex for most participants. However, the current version of Empatica E4 claims to have improved automatic Bluetooth streaming of the data to the cloud, which if robust, would greatly reduce the burden associated with physiology sensing.

The acceptability of active sensing methods holds great clinical utility. Active personal sensing methods, such as EMA, offer unique insights into patient experiences, thoughts, and feelings that cannot always be captured accurately or comprehensively by passive methods. Self-reported EMA, in particular, seems likely to play a role in risk monitoring and other similar clinical applications. Thus, we were encouraged to find that even with a relatively high active burden of 4 times daily surveys, EMA was acceptable to participants, as assessed via self-report and behavioral adherence.

### Important Individual Differences in Subjective Perceptions Exist Both Within and Across Personal Sensing Methods

In this study, we included a second and more novel daily active sensing method, audio check-ins. These audio check-ins have great potential as a rich source of information about participants’ daily experiences. Natural language processing of transcripts of their check-ins can provide a novel window into their thoughts [79-82]. These audio check-ins provided participants with the opportunity to share more openly and candidly (ie, without close-ended questions) their thoughts, feelings, and progress toward recovery without being limited to researcher-selected prompts. Analyses of the acoustic characteristics of their check-ins may yield independent measures of their affective state [83,84], including the potential for measuring affect outside the participant’s conscious awareness.

Unfortunately, overall participant adherence to the daily audio check-ins was relatively low (on average, 54.3% of audio check-ins were completed) and 1.9% (3/154) of the sample did not complete any check-ins throughout their entire study period. Participants’ free-response evaluations of this method highlighted some concerns that could be addressed in the future to increase adherence (eg, timing of the check-ins, technical issues with recording and sending check-ins, and use of the same prompt for all check-ins). However, privacy issues related to recording the audio check-in were also reported by many participants.

These privacy concerns represent an inherent challenge to using this method as implemented, but accommodations could be made to gather some, if not all, of the same information. For example, using less-frequent prompting with wider time completion windows (ie, a weekly audio check-in) may increase individuals’ ability to find a private moment. In addition, allowing individuals to type their response as an alternative completion method could assuage concerns. This alternative would prevent acoustic analysis, but it would still permit natural language processing of open-ended responses. These accommodations could encourage greater adherence among those who completed few or no audio check-ins, as well as individuals who missed check-ins sporadically because of privacy concerns. Finding ways to assuage privacy concerns and accommodate individual preferences may be useful, as many other participants valued and believed that they benefited from recording these daily audio check-ins.

Consistent with this somewhat polarized evaluation of the audio check-ins, a more nuanced consideration of the distribution for adherence across participants suggested that it was somewhat bimodal. Participants tended to adhere well or poorly to this method.

More broadly, the participants’ self-reported perceptions were only moderately consistent across the different sensing methods. This can be observed in the moderate ICCs (and bivariate correlations) across the methods for each self-reported item. In other words, high dislike ratings for 1 sensing method by a specific participant did not strongly indicate that the same participant would also dislike the other sensing methods. This is also true for the “ratings of interference” and “willingness to use for 1 year” items. Participants could dislike (or be unwilling to use) one method but not others. To the degree to which concerns are method specific, opportunities may exist to tailor sensing systems to user preferences. In other words, participants could opt out of the methods they deemed unacceptable but provide data for other sensing methods that were acceptable to them. For example, our behavioral adherence data suggest that some participants would not have completed the study if daily audio check-ins were required; however, they were willing to provide data via other personal sensing methods. Algorithms that use sensed data for clinical applications can then be developed for different combinations of the available raw data streams. Participants could be informed that personalized algorithms will likely perform better if given access to more raw data streams. This education will allow them to make an informed choice regarding the threshold they set for themselves to opt out and the potential consequences of not providing that data source. However, allowing them to opt out of some methods may increase the number of participants who will agree to provide sensed data.

### Benefits Likely Matter

The overall acceptability of personal sensing to research participants and patients is likely a function of both the perceived costs and benefits for these individuals [85-87]. However, we focused on measuring only perceived costs (eg, privacy and burden) associated with personal sensing because the benefits to participants from the sensed data collected in this research study were minimal. Participants were provided with modest financial incentives to complete the EMAs (US $25/mo) and to provide access to the 2 passively sensed raw data streams (US $10/mo for geolocation and US $15/mo for cellular communication logs with SMS text message content). These sensed data streams were not used to provide any clinical benefit to participants’ recovery in our study, although they hold great promise for use in machine learning algorithms that could predict lapses and deliver or tailor interventions to individual participants’ needs and recent experiences.

Monetary incentives are commonly used in research to provide a more favorable cost-benefit ratio surrounding specific methods or overall participation. Such monetary incentives are commonplace and recommended when using active personal sensing methods such as EMA [88]. However, the incentives to provide access to passively sensed geolocation and cellular communication in our study may have contributed to the acceptance of these methods and the success we had collecting these sensitive data from participants. This may be particularly true given the relatively low socioeconomic status of many of our participants. For example, the mean personal income for our participants was US $34,233, with 12.3% (19/154) of individuals reporting current unemployment and 25.3% (39/154) reporting an annual income below the 2022 federal poverty level.

Monetary incentives to increase the acceptability of personal sensing do not need to be limited to research settings. Incentives can also be used as a part of treatment or continuing care in clinical settings. For example, the use of monetary incentives or equivalents (eg, prizes) as part of a contingency management program is well established to support abstinence from alcohol or other drugs or adherence to treatments or other healthy behaviors [89-91]. If personal sensing proved useful for the treatment or ongoing support of patients’ recovery, similar incentives could be established to encourage patients to provide these sensed data.

Incentives may be less necessary in clinical settings when more direct clinical benefits from personal sensing are available. For example, research has suggested that privacy concerns associated with personal sensing may be reduced if participants perceive that they will benefit from the sensed data [6,51,87]. There was some evidence for this perspective in the free-response comments from our study participants as well.

We did not provide direct clinical treatment to the participants. Participants were given resources for alcohol treatment options upon request. In addition, although personal sensing methods were used solely for data collection, in this study, participants may have experienced some clinical benefits from them (eg, via reflection and accountability). However, the acceptability of personal sensing may be higher than that observed in our study if the sensing system was implemented as part of their direct treatment or continuing care during their recovery. Digital therapeutics are particularly well positioned to use sensed data to select, personalize, or time the delivery of interventions and other supports to improve clinical outcomes. Future research should evaluate the acceptability of personal sensing in contexts where its use directly benefits those providing the sensed data. In these contexts, benefits (eg, financial and clinical benefits) can also be explicitly measured. It may even be possible to manipulate the benefits from personal sensing across participants to evaluate their contribution to acceptability more rigorously.

### Trust Likely Matters

Trust is also likely to affect the overall acceptability of personal sensing data, which are inherently private and sensitive. Acceptability may depend on who uses personal sensing and who has access to raw and processed data [50,87,92-94]. The available evidence suggests that people are more comfortable sharing private, sensitive information with researchers and their physicians and less comfortable sharing information with family members, electronic health record databases, and third-party apps and websites [92-94].

The research setting may come with relatively greater trust because of the high level of transparency regarding the risks and protection measures associated with obtaining informed consent. Some protections may only be feasible for research as well. For example, National Institutes of Health (NIH)–funded research that collects identifiable, sensitive information is automatically issued a Certificate of Confidentiality that prohibits disclosing this information to anyone not connected to the research, except when the participant consents or in a few other limited situations. The Certificate of Confidentiality can also be requested for similar research not funded by the NIH. We saw evidence of the role of trust in the free-response comments from our participants. Our participants appeared to recognize and appreciate the protective measures taken to secure their data.

Implementations of personal sensing for treatment inside and outside clinical care settings [34] will need to carefully consider how to establish similar, high levels of trust. Clinical applications of personal sensing may sit at an intersection of sharing data with physicians (with which individuals tend to be comfortable) and with electronic health record databases and apps (with which individuals tend to be less comfortable) [93,94]. For example, it may be necessary to protect against the subpoena of sensitive information in civil and criminal proceedings. Patients will also likely need to be assured that sensed data used for their clinical care will not be shared with their health insurance provider with associated risks related to higher insurance premiums or dropped coverage. These issues of data access and unauthorized secondary use of otherwise private information are often cited as concerns regarding personal sensing [87,95].

Regardless of the setting, trust may be lower in stigmatized groups that could otherwise benefit from personal sensing. For example, individuals with mental illness still experience substantial stigma that could impede their willingness to share personal, sensitive information with researchers or clinical care providers [96-99]. In fact, we focused on individuals with AUD in this study to evaluate the acceptance of personal sensing methods in a population that we expected might have barriers associated with trust. Nevertheless, trust may still be lower among individuals with other substance use disorders that involve illegal drug use. However, many of our participants reported ongoing use of drugs other than alcohol throughout the study (75/154, 48.7% reported illicit drug use in the past month) as expected, given the high rates of polysubstance use among individuals with substance use disorders. Furthermore, we have had promising preliminary success in recruiting patients with opioid use disorder for an NIH-funded study on personal sensing in this population [100]. This suggests that our results regarding the acceptance of personal sensing may be generalized across substance use disorders.

Trust and related privacy concerns may also be more difficult to overcome in historically marginalized groups that have experienced systemic racism and other stigmas or exclusions [101]. These individuals may find it more difficult to achieve privacy in their daily lives, and they may hold very different perspectives on the costs versus benefits of surveillance in the context of personal sensing or more generally. Unfortunately, our sample was not diverse with respect to race and ethnicity. Future research on personal sensing must specifically recruit for such diversity to better understand its acceptance in racial and ethnic minority communities. We have learned from this study and adjusted our recruiting efforts accordingly to recruit a sample that is more diverse with respect to race, ethnicity, and geographic region for our ongoing personal sensing project with individuals with opioid use disorder.

### Feasibility Is a Function of More Than Participant Perceptions of Acceptability

User acceptance of personal sensing methods is necessary but not sufficient to expand the use of these methods in research and clinical implementations. A variety of other key issues may facilitate or present barriers to the wider use of personal sensing. These include cost and accessibility, stability over time, and the utility of personal sensing relative to other more traditional methods.

The smartphone itself is arguably the best available sensing system at present. Currently, smartphones contain numerous sensors and other raw data streams that can be used for personal sensing. In our study, we took advantage of GPS and other location services to track geolocation and used the microphone for daily audio check-ins. We accessed smartphone calls and SMS text message logs for communication metadata and message content, respectively. The smartphone also provided a convenient platform to collect self-reported EMA.

In addition, smartphones provide a relatively accessible platform for personal sensing. Despite their high cost, 85% of adults in the United States already own a smartphone. Equally important, this level of ownership is relatively consistent across race and ethnicity, geographic regions (eg, urban, suburban, and rural), and income levels [3]. Furthermore, people with substance use disorders generally have high rates of mobile technology use [102]. Notably, only 11 (6.5%) of the 169 eligible participants in our study did not already own a contemporary smartphone. In a research setting, we were able to provide individuals with a smartphone if they did not already have one. Similar to monetary incentives, this practice need not be limited to research; smartphones can be provided to permit personal sensing–based clinical support.

Personal sensing can also be performed using wearable devices or other sensors outside the smartphone. We used Empatica and Beddit systems to sense physiology and sleep, respectively. The use of watches (eg, Apple Watch) and wristbands (eg, Fitbit) for sensing activity and some physiology parameters is also increasing [18,103]. However, some of these systems can be expensive, and unlike smartphones, none have been adopted widely enough to assume that most users will already own the said devices. For research applications, this limitation can be overcome by providing the hardware to participants as needed. Although it is not impossible to do the same in clinical settings, the large number of patients who would require this technology may either limit or increase the cost to scale the sensing system.

Both research and clinical applications of sensing systems require some guarantee that the hardware and software will remain available and supported for the duration of the intended use. Unfortunately, there are currently high levels of churn among the companies that support these systems, given the rapid innovation occurring at this time. We collected data for approximately 2.5 years between 2017 and 2019. During this time, Apple bought the company that developed the Beddit Sleep Monitor and discontinued support for previous users. Apple reintroduced the sleep sensing system for iPhone users in late 2018 but discontinued it again in early 2022. Therefore, we were able to collect sleep sensing data from fewer than half of our research participants.

During this same data collection period, there was also a churn in the software that we used for sensing geolocation. We used the Moves app at the start of the study but needed to switch to the FollowMee app when Facebook acquired the company that developed Moves and discontinued its support. However, this software churn was less disruptive because both apps relied on smartphone sensors to acquire the raw geolocation data stream. This suggests yet another reason to prefer systems that make use of generic smartphone sensors rather than proprietary hardware.

High rates of churn can also affect the perceived acceptability of the software. For example, it could be inconvenient to have to adapt to frequent changing of app platforms. In addition, software may be left unmonitored for periods, leaving new bugs unresolved. In our sample of participants, we observed how frustrating technological issues were.

### Limitations and Future Directions

Conclusions regarding the acceptability of these sensing methods may not be generalizable beyond the 3-month study duration. Although 3 months represents a notable extension beyond the existing literature on personal sensing in clinical populations, it is likely not long enough, given the chronic-relapsing nature of alcohol and other substance use disorders. One potential concern is that the initial novelty of sensing may lead to overestimated adherence and subjective ratings of acceptability that is not sustained for longer periods [104].

This 3-month period also constrains our conclusions regarding the acceptability to people early in recovery. It is possible that acceptability ratings will vary depending on where someone is in their recovery phase. This may also be amplified when potential benefits are considered. For example, someone who has achieved long-term stability in their recovery could find that the costs of personal sensing (eg, data sharing and high effort demands) do not outweigh the benefits (eg, daily reflection on sobriety and potential for increased lapse risk awareness). It is important for future studies to extend study length and incorporate other facets of acceptability (eg, benefits) to account for these possible effects. In an ongoing study of people with opioid use disorder, we requested that participants use various active and passive personal sensing methods for 1 year [14]. In addition, future research could compare acceptability ratings for personal sensing methods between people with and without a substance use disorder.

Future studies should also examine the nuances of behavioral measures of acceptability. Our study was limited in the conclusions we could draw about adherence to passive personal sensing measures. All our research participants (154/154, 100%) provided some geolocation and cellular communication data and all but one of our research participants (153/154, 99.4%) provided SMS text message content data. However, we cannot know if and how frequently participants were choosing to selectively delete SMS text messages or turn their geolocation off. In addition, we have limited information on the reasons for participant discontinuation before enrollment. Only 1 participant did not consent to participate at the time of screening. However, the attrition between screening and enrollment could reflect some reservations about the personal sensing methods and the study as a whole. That said, we do not believe our attrition rates between these 2 visits to be unusually high for our target sample (ie, people early in recovery from AUD).

Our self-report acceptability questions were developed in house. Therefore, our results should be interpreted in light of our specific questions and settings. For example, we asked participants if they would be willing to use a personal sensing method for 1 year to help with their recovery. This could imply that there would be a clinical benefit to using the method for 1 year and may factor in their judgment of acceptability. These questions have not been previously used in other research settings. Although we attempted to minimize social desirability effects and encourage feedback (eg, deidentified self-report surveys submitted through a web-based survey platform), it is possible that these effects are built into our results. Nonetheless, it should also be acknowledged that the study conclusions are based on both these self-report measures and behavioral indices.

Finally, although our results suggest that clinical samples of people with AUD may find these personal sensing methods acceptable, more research is needed to test the acceptability of these methods in future applied-clinical settings, where issues of costs, benefits, and trust may differ meaningfully in complicated ways from the research context. Future studies should also examine how these personal sensing methods might be perceived by people with recovery goals other than abstinence. No technical reasons prevent personal sensing from being applied to alternative recovery goals (see the studies by Bae et al [28] and Walters et al [29] for examples of predicting current and imminent drinking episodes, respectively, in people without a goal of abstinence). In addition, it must be acknowledged that the individuals in our study agreed to participate in a research study on mobile health and were financially compensated for their time. It is unclear how these individuals and the research setting may differ from those seeking to use these methods in future clinical settings, where costs, benefits, and trust may all weigh differently on their decisions to engage with the sensing system.

### Conclusions

This study demonstrated the acceptability of several personal sensing methods. These methods were acceptable (1) over a longer period than has previously been assessed, (2) across active and passive methods, (3) despite the sensitivity of the data, (4) among individuals with AUD who may have greater privacy concerns, and (5) without explicit clinical benefits to the participants. These findings suggest that personal sensing methods are poised as accessible, feasible avenues to collect data about individuals to be used for clinical applications. More work is needed to determine the predictive utility of the data that can be collected via personal sensing, but our study shows that this work will be worthwhile to pursue.

Personal sensing is acceptable, and the technology to collect it (namely, the smartphone) is widely accessible. Personal sensing can make digital therapeutics—smartphones and web-based apps that provide mental health care—smart. These methods can personalize care for individuals such that they receive the specific interventions and support they need at the time they need them. Smart digital therapeutics can be scaled widely to provide treatment to the overwhelming majority of individuals who do not currently receive mental health care. They can reach those who have historically been excluded from or have otherwise faced barriers to care. With personal sensing powering digital therapeutics, we are positioned for a paradigm shift in mental health care. This study brings us one step closer to this goal, ensuring that the methods we hope to use to revolutionize care are acceptable to patients who will use them.

## Appendix

Multimedia Appendix 1 Transparency checklist.

Multimedia Appendix 2 Supplemental analyses.

Multimedia Appendix 3 Acceptability survey.

## Abbreviations

AUD: alcohol use disorder
EMA: ecological momentary assessment
ICC: intraclass correlation coefficient
NIH: National Institutes of Health
