# Supplementary material for: Acceptability of Personal Sensing Among People With Alcohol Use Disorder: Observational Study
Source: JMIR Mhealth Uhealth. 2023 Aug 28;11:e41833. doi: 10.2196/41833 (PMC10495858; doi:10.2196/41833)
Supplement: Multimedia Appendix 1 [file mhealth_v11i1e41833_app1.docx]

# Transparency Report 1.0 (full, 36 items)

Manuscript Title: Acceptability of Personal Sensing among People with Alcohol Use Disorder

Authors: Kendra Wyant, Hannah Moshontz, Stephanie B. Ward, Gaylen E. Fronk, John J. Curtin

Corresponding author’s email address: [jjcurtin@wisc.edu](file:///C:\Users\khefner\AppData\Local\Chrome\Downloads\jjcurtin@wisc.edu)

Link to Project Repository: <https://osf.io/cjsvk/>

###

### Preregistration Section

1. Prior to analyzing the complete data set, a time-stamped preregistration was posted in an independent, third-party registry for the data analysis plan. **No**

**Comments about your Preregistration**

We collected these data beginning in early 2017, prior to the point that our laboratory was routinely pre-registering analysis plans.

### Methods Section

**The manuscript fully describes…**

1. the rationale for the sample size used (e.g., an a priori power analysis). **Yes**
2. how participants were recruited. **Yes**
3. how participants were selected (e.g., eligibility criteria). **Yes**
4. what compensation was offered for participation. **Yes**
5. how participant dropout was handled (e.g., replaced, omitted, etc). **Yes**
6. how participants were assigned to conditions. **N/A. There are no conditions.**
7. how stimulus materials were randomized. **N/A.**
8. whether (and, if so, how) participants, experimenters, and data-analysts were kept naive to potentially biasing information. **N/A. This is an observations study that does not include analysis of group or manipulations. There were no study conditions to blind.**
9. the study design, procedures, and materials to allow independent replication. **Yes**
10. the measures of interest (e.g., friendliness). **Yes**
11. all operationalizations for the measures of interest (e.g., a questionnaire measuring friendliness). **Yes**

### Results and Discussion Section

**The manuscript…**

1. distinguishes explicitly between “confirmatory” (i.e., prespecified) and “exploratory” (i.e., not prespecified) analyses. **There were no pre-registered, “confirmatory” analyses in this study. The analyses in the study are primarily descriptive.**
2. describes how violations of statistical assumptions were handled. **No**
3. justifies all statistical choices (e.g., including or excluding covariates; applying or not applying transformations; use of multi-level models vs. ANOVA). **Yes**
4. reports the sample size for each cell of the design. **Yes**
5. reports how incomplete or missing data were handled. **Yes**
6. presents protocols for data preprocessing (e.g., cleaning, discarding of cases and items, normalizing, smoothing, artifact correction). **Yes**

### Data, Code, and Materials Availability Section

**The following have been made publicly available…**

1. the (processed) data, on which the analyses of the manuscript were based. **Yes**
2. all code and software (that is not copyright protected). **Yes**
3. all instructions, stimuli, and test materials (that are not copyright protected). **Yes**
4. Are the data properly archived (i.e., would a graduate student with relevant background knowledge be able to identify each variable and reproduce the analysis)? **Yes**
5. The manuscript includes a statement concerning the availability and location of all research items, including data, materials, and code relevant to the study. **Yes**
