# Supplementary material for: Acceptability of Personal Sensing Among People With Alcohol Use Disorder: Observational Study
Source: JMIR Mhealth Uhealth. 2023 Aug 28;11:e41833. doi: 10.2196/41833 (PMC10495858; doi:10.2196/41833)
Supplement: Multimedia Appendix 2 [file mhealth_v11i1e41833_app2.pdf]

Multimedia Appendix 3: Supplemental Analyses

Acceptability of Personal Sensing among People with Alcohol Use Disorder

Kendra Wyant, Hannah Moshontz, Stephanie Ward, Gaylen Fronk, John Curtin

2023-08-09

Contents

Physiology Analysis . . . 2
Time Analysis . . . 3
Audio adherence distribution . . . 4
Adherence over time . . . 5
Active vs Passive Methods on Dislike . . . 6
Analysis on willingness to use EMA one time daily . . . 7
Active vs Passive Methods on Willingness to Use for 1 Year . . . 8
Uncorrected pairwise comparisons among personal sensing data streams . . . 9
Participant free-responses to each personal sensing measure . . . 10
- Audio Check-in . . . 10
- Geolocation . . . 15
- Cellular Communications and Text Message Logs . . . 20
- Sleep Quality . . . 25
- EMA . . . 28

Sample participant free-responses mapped onto each theme . . . 33
Group differences between continued and discontinued participants . . . 36
Reasons given for participant discontinuation . . . 37

Purpose: This file is the supplemental analysis script for *Acceptability of Personal Sensing among People with Alcohol Use Disorder*. It includes all supplemental figures, tables, and statistical analyses.

Physiology Analysis

We initially monitored participants physiology using an early version of the Empatica E4 wristband monitor. Due to a complicated sensing platform and software bugs we discontinued the wristband after 9 participants. Below are the self-reported acceptability ratings (interference, dislike, willingness to continue for 1 year) for this sensing method from the nine participants who used it (Figure S1).

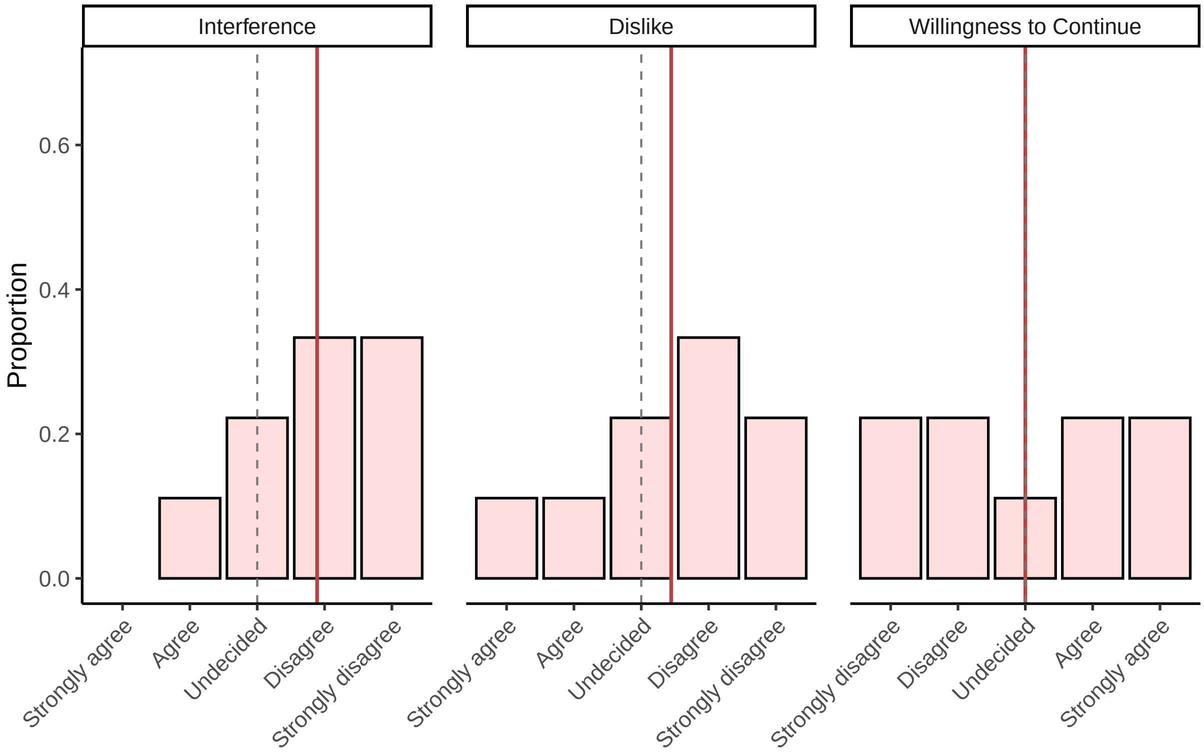

Figure S1: Subjective Acceptability of Physiology Personal Sensing Method. X-axes are ordered to display higher acceptability on the right side. $N = 9$. Solid red line represents the mean and dashed line represents the neutral midpoint (undecided).

Time Analysis

We examined acceptability ratings over time for participants who completed the study through follow-up 3 ($N = 133$). We see average acceptability ratings for each personal sensing data stream are relatively stable across time (Figure S2).

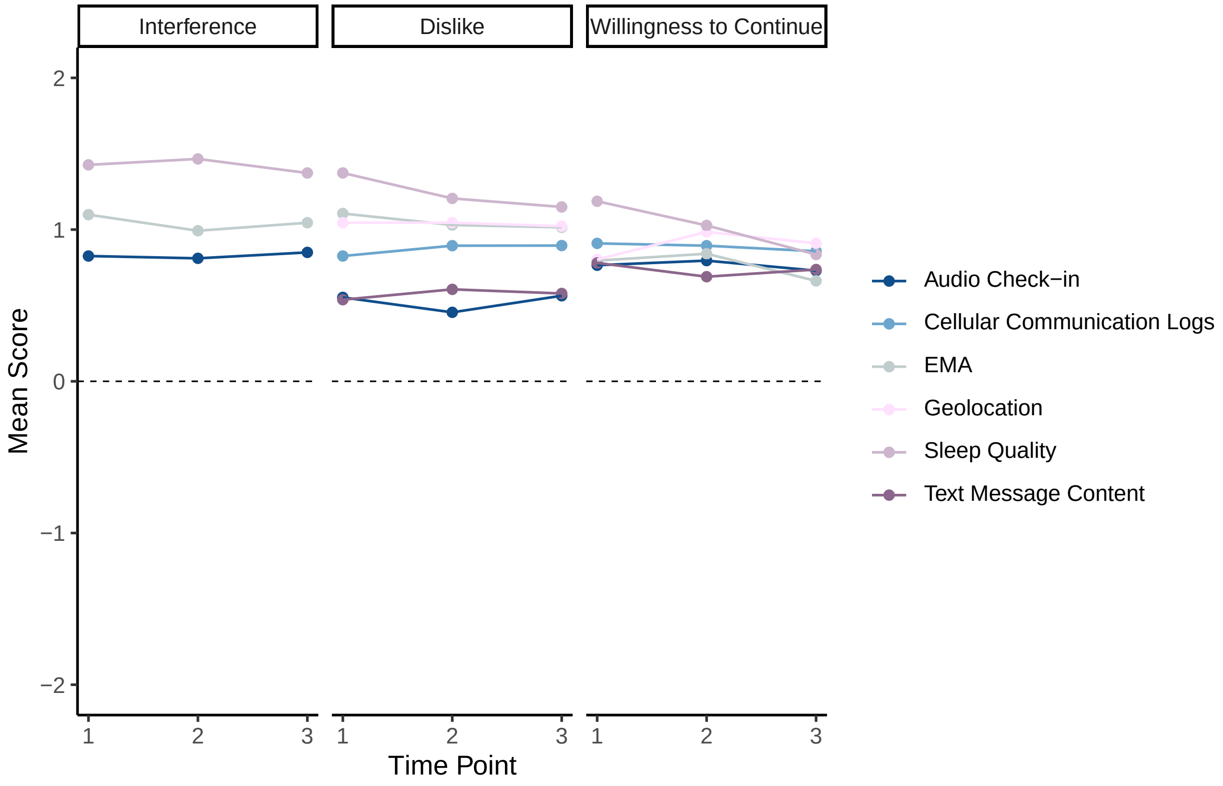

Figure S2: Acceptability over Time by Personal Sensing Data Stream. Participant ratings ($N = 133$) for each subjective measure of acceptability at three different time points one-month apart. Sleep quality $N = 87$. X-axes are ordered to display higher acceptability on the right side. Dashed line represents the neutral midpoint (undecided).

Audio adherence distribution

Participants tended to either comply well or very poorly with the audio check-in. This appears to be consistent with subjective evaluations (i.e., free-response comments) of acceptability (Figure S3).

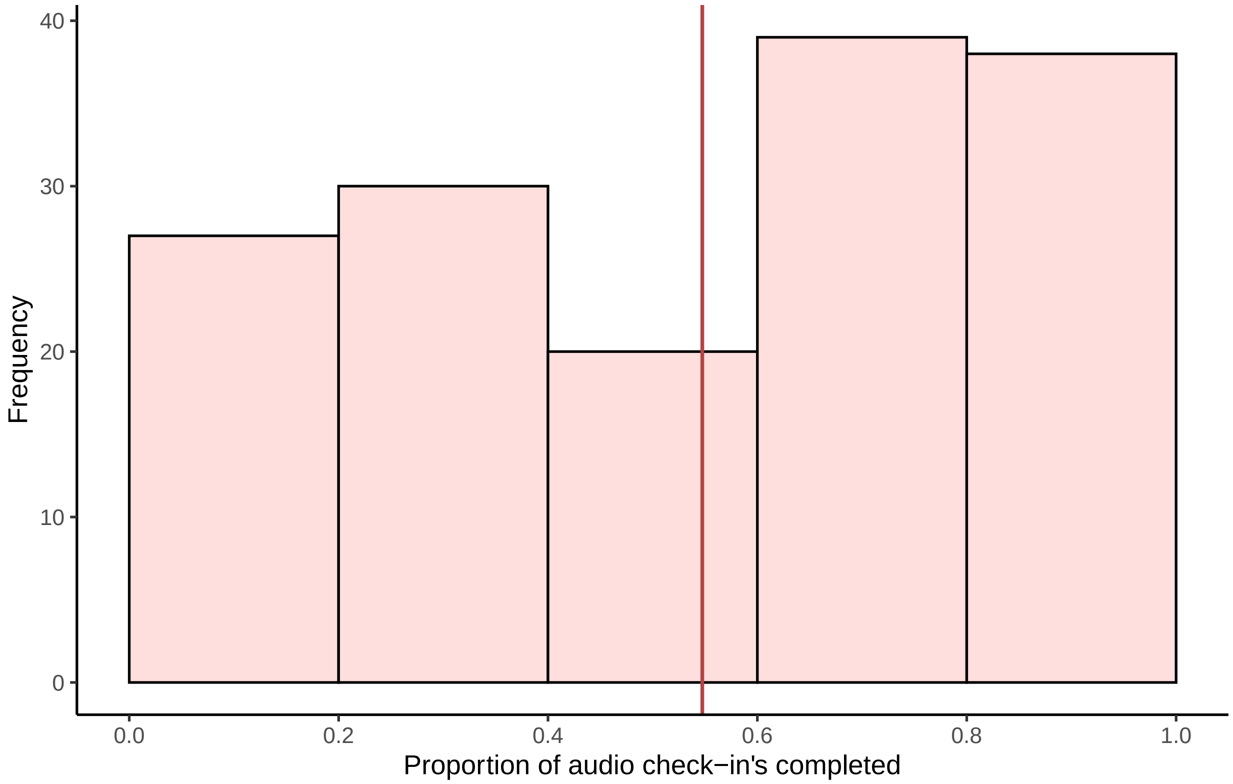

Figure S3: Audio Adherence by Participant. $N = 154$. Solid red line represents mean adherence.

Adherence over time

In the manuscript we report adherence over time on study (i.e., week) for our whole sample (N = 154). It is possible that adherence averages are lower for participants who drop out of the study (N = 21) compared to those who complete the full 3 months of the study (N = 133). We tested this by running independent samples t-tests on mean adherence scores for the two groups.

A t-test revealed that participants who did not complete the study on average had significantly lower adherence for the 4x daily EMA (M = .66, SD = .19) compared to participants who completed the study (M = .82, SD = .14), t(152) = -4.6, P < .001. Additionally, participants who did not complete the study on average had significantly lower adherence for a 1x daily EMA (M = .86, SD = .14) compared to participants who completed the study (M = .95, SD = .09), t(152) = -4.4, P < .001. Finally, a third t-test revealed that participants who did not complete the study on average had significantly lower adherence for the daily audio check-in (M = .36, SD = .23) compared to participants who completed the study (M = .58, SD = .29), t(152) = -3.2, P < .01.

Below we plot the total adherence with EMA (4 prompts per day), EMA (1 prompt per day), and the daily audio check-in over time for participants who completed the study (Figure S4).

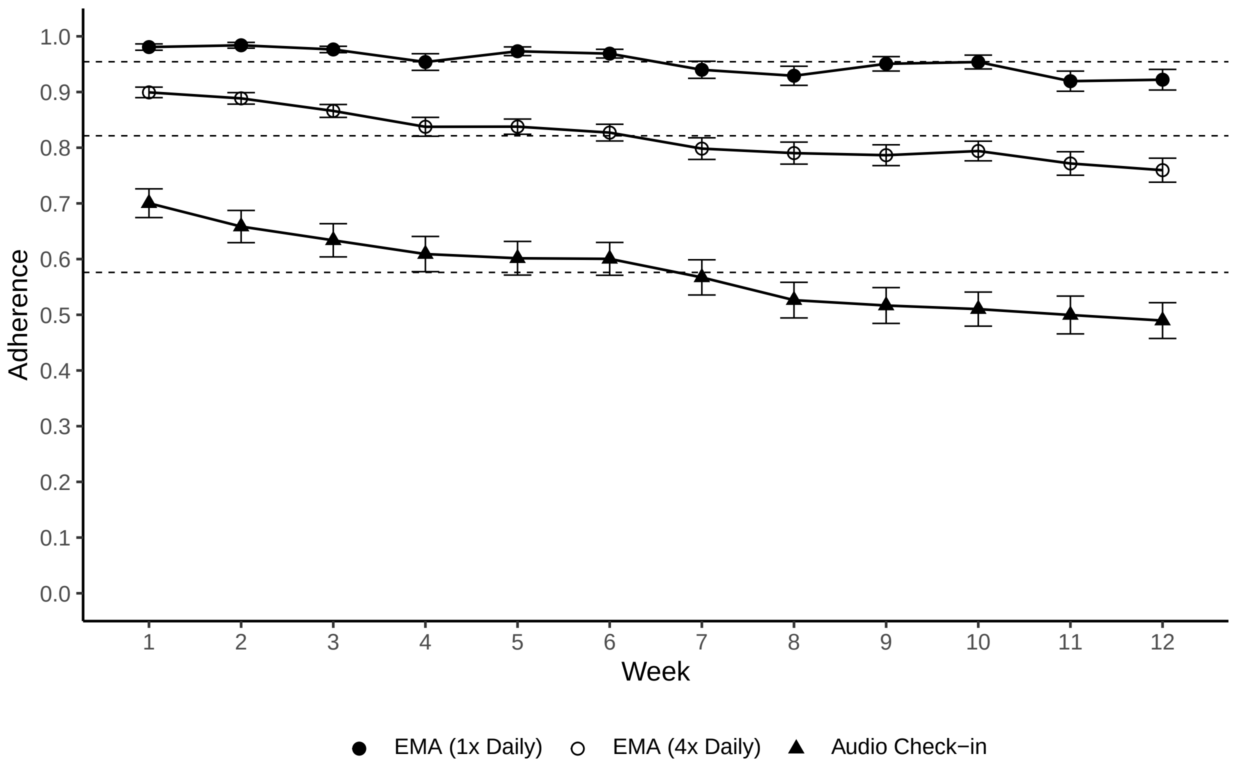

Figure S4: 1X Daily EMA, 4x Daily EMA, and Audio Check-in Adherence over Time for Participants who Completed the Study. Average adherence by week for participants who completed the full 3-month study (N = 133). Dashed line represents overall mean adherence.

Active vs Passive Methods on Dislike

We also assessed the effect of active effort on dislike ratings (Figure S5). We conducted a paired samples t-test to compare the average dislike for active (audio check-in, EMA) vs. passive (geolocation, cellular communication logs, text message content) methods.

Participants did not significantly differ in their dislike of active vs. passive methods, $t(153) = 1.21$, $P = 0.23$, $d = 0.1$.

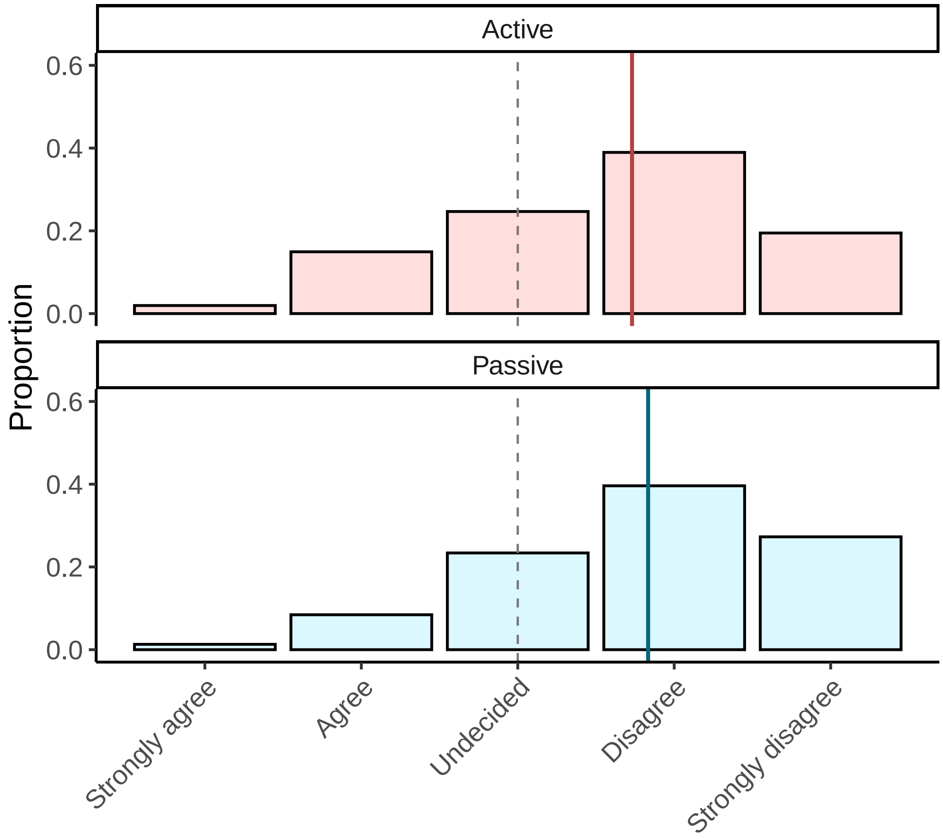

Figure S5: Average Dislike by Active vs. Passive Methods.
Notes: X-axes are ordered to display higher acceptability on the right side. Active methods (displayed in red) represent an average of audio check-in and EMA. Passive methods (displayed in blue) represent an average of geolocation, cellular communication logs, and text message content. Solid red or blue line represents the mean and dashed line represents the neutral midpoint (undecided). Participants did not differ significantly in their dislike of active vs. passive methods. $N = 154$.

Analysis on willingness to use EMA one time daily

Participants reported on their willingness to continue to use an EMA method that was 1X daily for 1 year. This method was hypothetical in that although they were asked about a 1X daily EMA they were expected to do 4 daily EMAs.

A one sample t-test revealed that the mean willingness score (depicted as the solid red line in Figure S6) was significantly more acceptable than 0 (gray dashed line indicating undecided), $t(153) = 15.21$, $P < .001$.

Additionally, we ran a paired samples t-test to compare participants' willingess to use a 1X daily EMA for 1 year compared to a 4X daily EMA. We found that Participants were significantly more likely to be willing to complete EMA's 1X daily ($M = 1.26$, $SD = 1.03$) compared to 4X daily ($M = 0.64$, $SD = 1.22$) for 1 year to help their recovery, $t(153) = -8.32$, $P < .001$.

Below is the distribution of responses for this question (Figure S6).

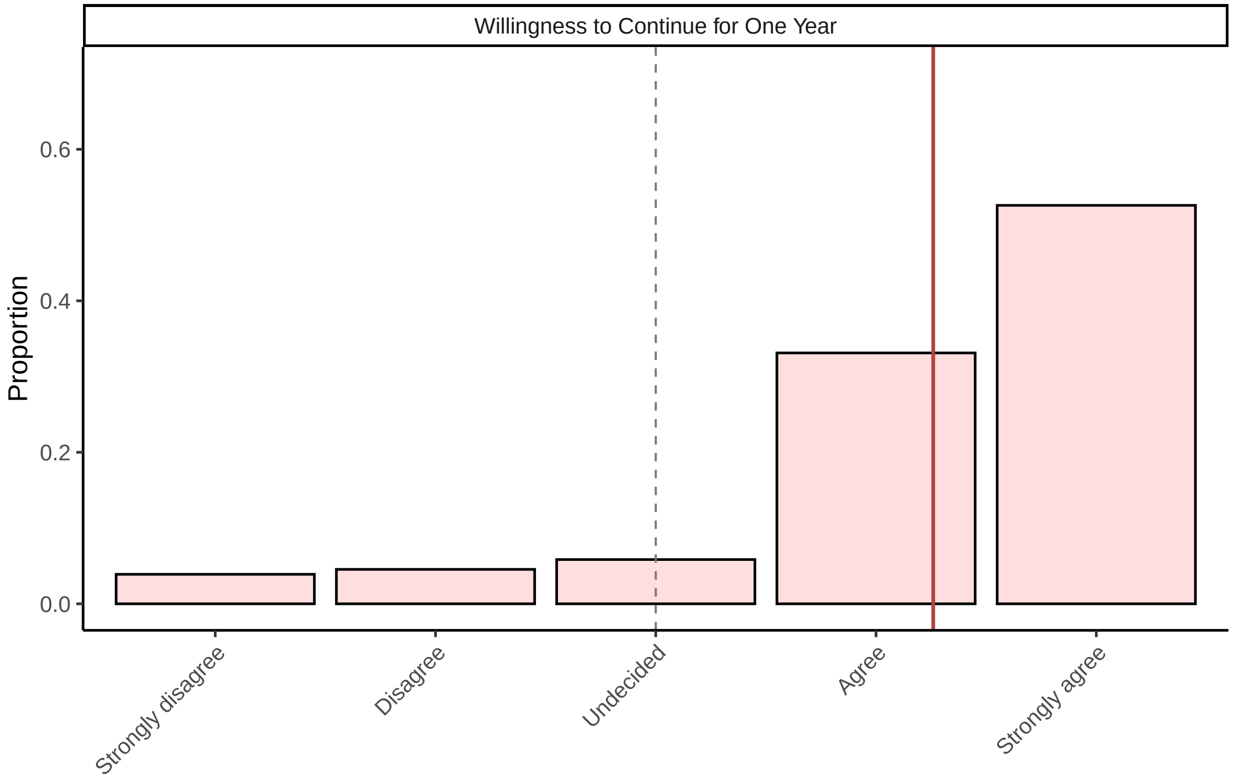

Figure S6: Willingness to Complete a 1X Daily EMA for One Year. $N = 154$. Solid red line represents the mean and dashed line represents the neutral midpoint (undecided).

Active vs Passive Methods on Willingness to Use for 1 Year

We also assessed the effect of active effort on willingness ratings (Figure S7). We conducted a paired samples t-test of the average willingness to use for 1 year for active (audio check-in, EMA) vs. passive (geolocation, cellular communication logs, text message content) signals.

Participants reported higher acceptability with respect to willingness for passive data streams ($M = 0.8$, $SD = 1$) relative to active data streams ($M = 0.7$, $SD = 1.1$), $t(153) = 2.12$, $P = 0.04$, $d = 0.17$.

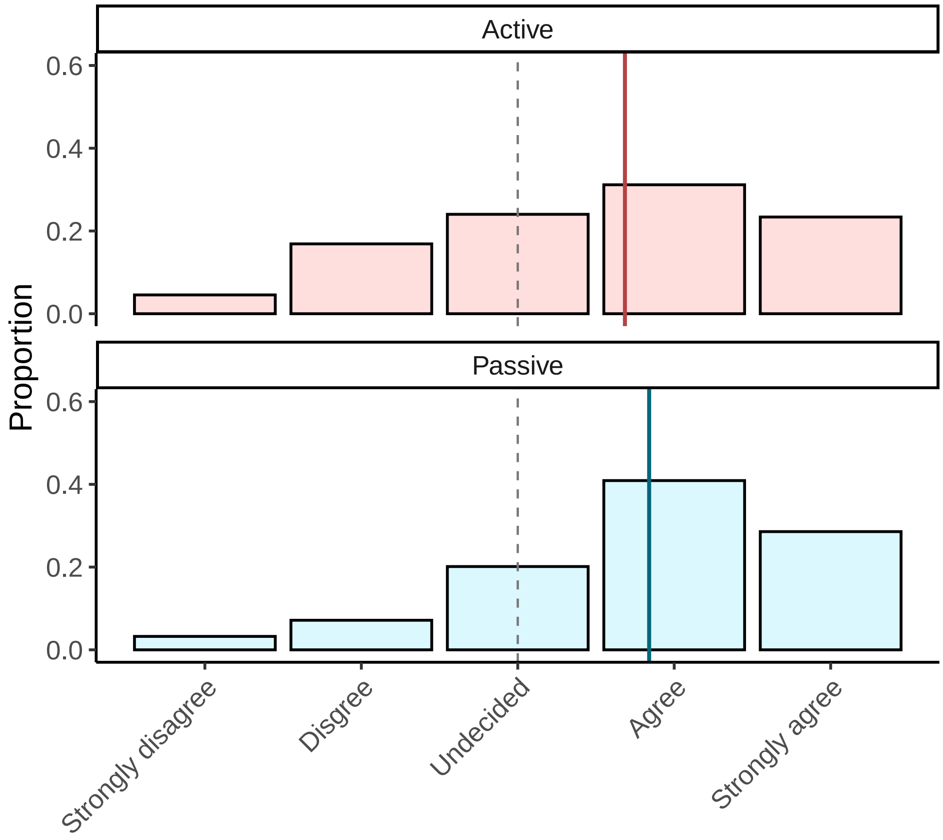

Figure S7: Average Willingness to Continue for 1 Year by Active vs. Passive Methods.
Notes: X-axes are ordered to display higher acceptability on the right side. Active methods (displayed in red) represent an average of audio check-in and EMA. Passive methods (displayed in blue) represent an average of geolocation, cellular communication logs, and text message content. Solid red or blue line represents the mean and dashed line represents the neutral midpoint (undecided). Participants reported on average significantly higher acceptability with respect to willingness to continue using for 1 year for passive compared to active methods. $N = 154$.

Uncorrected pairwise comparisons among personal sensing data streams

Table S1 contains all possible combinations of pairwise comparisons among active and passive personal sensing data streams.

Table S1: Uncorrected Pairwise Comparison P Values for Pairs of Personal Sensing Data Streams across the Three Measures of Self Reported Acceptability

Measure	Audio Check-in	EMA	Sleep Quality	Geolocation	Cellular Communication Logs
Interference					
EMA	.25				
Sleep Quality	< .001***	< .001***			
Dislike					
EMA	< .001***				
Sleep Quality	< .001***	.30			
Geolocation	< .001***	.56	.59		
Cellular Communication Logs	.001***	.63	.15	.29	
Text Message Content	.56	.002**	< .001***	< .001***	.01**
Willingness to Continue					
EMA	.47				
Sleep Quality	.46	.18			
Geolocation	.14	.03*	.60		
Cellular Communication Logs	.42	.13	.97	.50	
Text Message Content	.96	.44	.49	.15	.44

Note:

Sample size for all personal sensing measures is 154 except for the sleep quality which had a sample size of 87.

* $P < .05$

** $P < .01$

*** $P < .001$

Participant free-responses to each personal sensing measure

We obtained participant's free-response comments for each personal sensing method as a qualitative index of acceptability. Below are the raw participant responses for each method (Tables S2-S6).

Audio Check-in

Table S2: Participant Free Response Comments about Audio Check-in ($N = 154$)

Comment
Was easy and almost kind like check in
Very interesting and 'em lighting.
This was my favorite part of the study. It helped me to set a good intention towards my recovery however I think it may have been more useful at the end of the day. You probably would have gotten better info at the end of the day
This tool helped me stay focused on my up and down feelings throughout the day. The one thing that would be nice is that at the end of the night it would be nice to be able to have a overview of feelings
Same feeling as with the general survey. This question helps me understand where I stand in my recovery and serves as a reminder for abstinence.
They were very helpful
They were easy for me
These were amusing to complete. It was nice that it was both brief and daily.
There in conjunction with text messages were a great reminder of my sobriety
The question helped me stay focused
The daily checkins become more difficult to complete as time went on. Specifically responding to how i was feeling in my recovery became more of a non issue as i worked to enhance the rest of myself
Reminds me of the goal
On some days, the check in with you may have been the most significant communication i had with anyone.
Nice to speak rather than type through the check in. Seemingly less direct information related to recovery was shared
I had no problem with it and liked starting my day with it.
It's nice to reflect on the noteworthy parts of my day
This wasn't an issue, unless I was constantly surrounded by people, or if I didn't do it right away and then forgot. It's a good way to center yourself and focus on what you are feeling that day.
It was good to think about how i was feeling each day, but also became a bit of a to do
It was again a question that keeps the person responding responsible for their actions
It made me pause for a second and really think about how my day was
It is a nice set of questions that you got. It was simple and easy.
Unfortunately my phone often cut me off at 10 seconds so that was frustrating but it helps you reflect on things
Fine it helps keep you on task
Again, it helped me stay focused on recovery

It helped me realize my emotions and I had something to answer to which helped keep me on track

It helped me be mindfull

I think that just this one survey would be beneficial

This part was a little repetitive but I found it mostly fine to do. I think it was most important for personal reflection throughout the day on my recovery process. I guess I think I probably gained more from this than you might have from my responses.

I think i got better at it especially if i did it while driving to work in the morning

I loved this part! It was like journaling kind of where i would discover things that were hidden in my subconscious. I have a recording app on my phone and i will continue doing the voice check in as a form of checking in with myself

I liked the question. It is early enough to not have to happen while working or trying to focus scattered thoughts in sea of change.

I liked the daily check omw it gave me a way to vent without being judged

I liked that they were open ended

I liked knowing someone would actually be listening to me and my voice and not just the survey questions.

The only negative part about the daily check in was my mom, who I live and work with, listening in while I was talking. I would have to find a time when she was preoccupied or away from me to talk freely. Other than that I found it very helpful to reflect on how my day was in general when it came to my urges and cravings.

These did not interfere and I felt it kept me more on task ton stay on track.

I enjoyed it actually. I kind of wish i had a copy of them to listen to!

Good , helped. Me not drink

Helped me to check in with myself

Has helped sobriet

I like it. Gives me something to do in the morning. The only thing I don't like is how the up one easily stops recording you if your finger comes off the record button for a micro second.

Encouraged recovery.

As stated previously I grew to like the opportunity to speak about how I felt.

Also miss check in. It was useful to think about each day and what happened.

Again helped keep recovery a priority and on my mind

I enjoyed the daily check more than all the other surveys. To be able to verbilize my feelings was the most best therapy. You should expand on this and have more verbal check-ins.

Yup

Was fine

This was fine.

They were goood

They were fine when i remembered to respond.

they were fine id change the Time they were sent to me. Maybe a evening audio

Positive

Positive

Not an issue, just sometimes got busy with life and forgot but overall no issues and kept me mindful

Not a big ssue

Not a big deal. No hassles.

If i was more of a diary type of person I'd probably be more into this kind of thing. Again no strong feelings i mostly just didn't know what to say.

No problems.

No inconvenience

No big deal, not a burden

Like the check ins

Kind of liked it

It's ok with me i don't have a problem with it

It's helpful

It was often difficult to find privacy to respond, though it would have been helpful to keep doing it.

It was ok

It was helpful

Inobtrusive.

I thought it was positive

I kept forgetting to do this until bedtime so it wasn't really positive or negative.

I had no is yes with the daily voice messages some times i just forgot

I enjoy it

I didn't mind the voice record as much

Good experience

Good experience

Fine. Short

All positive.

Would prefer a specific questions ion to answer or comment on

This was the most difficult part of the study for me. I eventually just stopped completing the audio journal because I just didn't have anything to say. It started to feel forced and unnatural

This one was harder to like because I felt like I needed privacy to complete it, which sometimes I don't have.

The daily recovery check in voice msging made me feel really uncomfortable

The audio app rarely worked with my phone

Technical difficulties with my phone made it hard to do those for a little while. Perhaps recommend deleting the message after sending to free up space?

Still not sure what this means. Audio survey had no question

Still hated these. Perhaps i would have liked it better if I had prompts to respond to.

Sometimes I wouldn't be able to do it because I was with people too much which was frustrating

Sometimes I didn't have time to send audio messages

Sometimes had trouble sending files.

Sometimes forget to do them or fall asleep too early

Quasi-redundant with questions.

Only inconvenient when I already was at work and needed a quiet/private place to speak.

Oftentimes found myself in public places. Hard to record and if I did it was hard to open up

Not sure how that helps me. No feedback from it so i wonder how helpful it is. Maybe if i had a specific question i was to answer.

Not a good look to be on your phone at work

My biggest thing about the voice survey is what's the point of it. Just to see if I'm alive and kicking in some coherent fashion?

Maybe right before bed, but hated it

It would be nice if it were an option. For example if I had something that was weighing on my mind or a new trigger event

The audio portion was terrible. It takes time out of your day where you have to completely switch locations just so you can do t in private. I don't like that people could hear me and the topic wherever and whenever so I stopped using it.

It seemed harder to do than text survey.

Kind of weird. If one had sonnet bf specific to talk about , it would be much better

If I didn't do it right away I'd always forget about it. I also have to keep privacy in mind since it's not something I'd like to complete within earshot of others.

I would prefer if the prompt changed over time. Recovery prompt was vague and old after the 4 months

I would have preferred at least two daily reminders to send it. I forgot to a few times.

I quit the call in when the online surveys became to grating bro complete.

I often couldn't think of anything to say so I would have found a less open-ended prompt useful

I never know what to say for the audio question.

I missed them sometimes because the regular survey and audio survey would come at different times and i would forget to go back and do the audio

I may not want to always mentally realize how often i feel bad

I just never really knew exactly what to say

I just could never remember if I'd done them and there's no way to see if you sent it.

I had a hard time keeping up with the daily check in but I wish that I had put more effort into this daily task. Many times I found myself reflecting on my recovery but I didn't put in the effort to make an audio recording of these reflections and I wish that I had

I found it hard to talk to a me home about what i was feeling

I found it hard to stay motivated to do it.

I felt like I needed to seek out a private spot to do the audio message. I would have much preferred to type the message.

I experienced issues with this from the very beginning and discontinued early on. I was unable to get the message beyond 8-10 seconds with close to 6 attempts each day. Was not worth the time or stress for me.

I did not do this part. I am not a fan of talking on/to a phone

I could never get into the routine of the audio check-in, it felt uncomfortable to me.

I am not much of a phone talker so I sometimes found it difficult to find words

Hard to find private time

Had trouble remembering to do it came to early in the day.

Generally disliked the open ended nature of these. Maybe provide a rotating prompt?

Easy to forget about if i didn't complete right away in the morning

Do to cut rant WiFi connections in my building i had to go out side to send voice mail.

Audio surveys were uncomfortable especially if there was no privacy to do them. They exemplified feelings of failure, defeat, and hopelessness for being able to achieve or maintain abstinence.

Again, didn't want to do it while drinking

Again being busy can lead to not completing the daily recovery

A little intrusive. One less a day would be ideal.

Was annoying at times but not so annoying that I didn't do it

These were annoying

Starting to become to much

Sometimes I forgot to call in

Same responses daily

Missed a few

It was brief but not my thing

I hate the audio check in!

I found the audio messages most challenging.

I forgot to check in daily

I dislike the audio check in

Got to be a burden as the study progressed.

Generally mildly negative

Again i just would forget or get side tracked.

See previous answer.

Same as previous

Same as last quetion

Same

Ok

Nuthing really

None

No thoughts

No comment

No

Na

Different

Geolocation

Table S3: Participant Free Response Comments about Geolocation ($N = 153$)

Comment
Neutral about this. Was okay to have done in the context of the study or for an app that would help me stay sober.
This didn't change where I chose to go.
This didn't affect my daily life at all. Unless the tracker randomly stopped tracking. I found that if I just left the app on in the background then it wouldn't quit tracking after phone updates.
Saving the information was great.
Location tracking is fun and useful for me personally in addition to the study.
It was Interesting to see my patterns and places
It never was a factor in my coming and going.
It is interesting to view how much time I spent at places and how many steps I have taken
It honestly stopped me from going places I know may be a trigger
If it helps me then I would do it
I thought it was kind of fun when i went someplace different and i would think of you guys helping me to stay sober
It was ok. I liked the fact I could shut it off if I wanted to
I don't mind it at all. It's sort of nice to know I'm being monitored. The state has been doing it for years anyway. I might as well be getting paid finally for having my location tracked.
I don't go out and about except for errands etc so no bother to me.
I don't mind being tracked since I appreciate the purpose of it.
Highly personal but if it helps okay
Helps my recovery and keeping me accountable
Not a problme, I have nothing to hide! Held me accountable for my actikns
Good for my recovery
No bother, easy to turn off
Don't mind if I can stay sober
Didn't even notice. That's the best part
Zero issues
Whatever. I don't care about it.
Wasn't a poblem
Wasn't a big deal.
Understandable but i usually have my location turned off on my phone
This does not bother me. I am too exhausted from the confusion of daily life to lie.
That didn't bother me. I feel like so much is tracked nowadays via our phones.
I'm a somewhat typical introverted, slightly paranoid, grump, so the idea of being tracked is automatically negative. That being said, if it's something that helps people that can be a really good thing.

Seems invasive in theory, but I've had no issues a it

Positive

Nothing to hide so not a problem.

Nothing to hide

Not intrusive at all.

Not an issue.

Not an issue

Not a problem

Not a bother

No worries.

No problem

No problem

No issues with tracking.

No issues occurred

No issues

No issues

No impact and if somebody is willing to gain sobriety there is no harm

No concerns

No concern

No big deal

Never gave it a thought

Never even thought about it

Mostly i did not think about it, my job with the cleaning service used similar software,.

Liked to use it

It's just normal

It's alright with me

It was ok

It was good

It was fine. I didn't really think about it.

It was diffrant

It doesn't matter. I didn't know what was happening with the location app. It was doing on its own.

It didn't bother me one way or the other

It didn't bother me

It did not affect me

Indifferent

I trusted the study group to not use my personal information for any other use.

I support voluntary data tracking for science

I personally don't mind having my location tracked, but i cod see how this would be a concern for others.

I never even thought about it.

I never could tell I was being tracked

I have nothing to hide.

I h number be nothing to hide or worry about as far as my locations.

I have nothing to hide

I have no issues with it

I had no is yes with this

I had no issues with my location being tracked

I got a new phone out of it

I forgot this was even happening most of the time. It did not interfere with my life.

I forgot I was being tracked so it was not a big deal to me

I felt all right having my location tracked. If it were used in a way to keep me from relapsing my feeling about it would be even more positive.

I don't see any negatives

I don't really notice this

I don't mind this

I don't mind it at all

I don't think about it.

I don't have strong feelings about this one way or the other. I never really even thought about it through the course of the study

I didn't think about it much.

I didn't really think about it. I just did whatI do.

I didnt mind that the location was being shared – so many apps use location nowadays anyway

I did„ care if someone tracks me

I didn't leave home very much and only turned location off about 2-3 times for short periods

I didn't even notice. Was totally unaffected.

I did not really think about it. I knew it was happening, but did not mind.

I did not mind having my locations documented, in which I didn't find any need to temporarily disengage throughout this whole process. However, My bigger problem with the tracker app. Was more the fact that my battery power was getting depleted so rapidly, sometimes within 2 hours post-full charge. I found myself charging my phone at least 5 times a day. And at times, this became very stressful, esp. When I would have a 100 percent battery when leaving my home for work and whatnot, sometimes forgetting my charger and chords, and trying to figure out how to get more juice so that my phone doesn't shut down in case I need to be reached for an emergency.

I believe the study is confienule

I am up for anything. This helps ,e to

I am interested to see how this data helps future addicts recover

I am fine with it since I'm not doing anything wrong anyways lol

I am an ACLU member, but i understand the utility for the study.

Having myself tracked didn't matter to me and wudnt matter to me

Have nothing to hide.

going to be using this afterwards.

Frankly i forgot about it

Found it helpful

Fine with me

Fine

Fine

Fine

Don't mind being tracked. No issues

Didn't really bother me

Didn't mind

Didn't give it a second thought.

Didn't even think about it

Didn't care or think about this

Didn't bother me or influence me at all

Did not affect me either way

Car insurance and life insurance companies already tracks my location so this was nothing new.

Being tracked doesn't bother me

As a geographer I like all mapping!

Also fine

All ok

The only issue was my phone loses service often. I have the oldest i phone available on the market so it is very unreliable. I never turned off my GPS location but quite often it would not record me going somewhere.

Sometimes it didn't ping my location very accurately

My only concern for privacy would be if the software was not secure and others not part of study staff could follow me.

It would be easy to just leave it at home if I wanted to go to a bar, etc

It sometimes tracked when simply stopped at an intersection.

I would never allow this in real life.

I still feel as though, at least theoretically, that I was being spied on, but in real life, I forgot about it. I can see how this could help some people, but I don't go to bars/clubs/etc. I don't see this helping me, personally.

I don't really like that you know everything I'm doing

Same, i don't feel comfortable with thebtracking

I don't entirely understand how I feel about this as, though it is a little creepy that I'm being tracked, i know that the study is doing it and there is no overall harm being done. A little invasive

I disliked how much the app drained my battery.

I am concerned with data privacy, and felt uncomfortable having my location actively tracked.

Feel weird about it because of today's times in our government. Just saying, feel like liberties are stripped away a lot so hard to trust anyone.

Cost me money

Undecided

Still not completely sure how I feel about it

Not fond of being tracked

Likely it would bother some people.

Kinda weird, but I understand

See last answer

Nuthing really

Nothing

None

No comment/neutral experience

No comment

No comment

No

Na

Na

Na

Meh.

Big data

Cellular Communications and Text Message Logs

Table S4: Participant Free Response Comments about Cellular Communication Logs and Text Message Content ($N = 153$)

Comment
This keeps me in tune with my feelings
Saving all my information was great and time saving.
Only for the sake of my recovery
Again, invasive in theory, no problems actually because of confidentality
Again. Never noticed so it was zero inconvenience whatsoever !
Loss of privacy for the sake of sorberity, okay I'll give in
No issues, like that they allow me to delete some items from the log if I wanted to.
I don't have much in secret so I guess, it ddoes not matter. Besides, it was easy and staff was doing everything. Thanks,
It did glean a highly amusing story for my memoir, so there's that. It is a huge invasion but my sobriety is paramount to my overall health.
It feels like an easy way to help me with my goal of staying sober. It helps me reflect on what I am doing and feeling.
If you're serious about recovery, some privacy is to be sacrificed.
If it helps recovery, fine with me,!
If i felt desperate in my recovery i would probably agree and appreciate that long term but i don't think it was necessary for me
I would recommend emails as well
I don't mind turning this info over and I trust the people running the study
I don't mind this at all either, as long as I know this information is protected and anonymous
Helps my recovery like i have someone to answer to
Having the opportunity to delete texts was nice even though I hardly ever did
I don't mind study staff having access to my texts and call logs. I'm not really that interesting and have nothing to hide. Especially if it helps keep me sober I'm all for it.
Didn't mind after I asked more about it
Didn't bother me since its protected
As long as my personal information was secure and only shared by study staff, and its collection could help in my recovery, I'm comfortable with it.
As long as it stayed strictly confidential.
Also a good insight for my recovery
Accountability keeps me honest
Wo agree to extend a year no problem
Whatever data helps you guys
Unobtrusive
This was not an issue. I had no concerns or stress about this,.

Positive you know if you mess up you don't feel better because of having your phone

Positive

The only way this impacted me at all was in that I couldn't clear my log history on a consistent basis, which I usually like to do. Otherwise, perfectly comfortable process.

Nothing to hide. My friends don't drink.

Not really noticeable

Not an issue. I'm not that interesting.

Not an issue

Not a huge comcern

Not a big deal

No problems with this

No problems

No problem

No problem

No negatives

No issues.

No issues

No issues

No issues

No issues

No concerns.

No concerns

Keep the tracking going.

It's ok i guess i have no worries

It wasn't bad

It was ok

It was ok

It was fine, but my messages are pretty irrelevant to my recovery

It was fine

It was fine

It was fine

It didn't really matter to me a lot, but it did feel somewhat invasive and extreme

Indifferent

I went about my days as i normally would and never thought about it.

I wasn't bothered by this for the most part.

I hope that this information does actually help with the outcome of this project. I'm not sure it will because of the hyper variability between each participant's messages.

I have nothing to hide.

I have no issues at all with tracking my calls

I hardly thought about it

I had no problem with this but would like to opt out when I am done with the study.

I had no issues with the tracking of my messages

I get a lot of unsolicited texts from advertisers and political campaigns.

I don't text or call a lot

I don't talk to that many people

I don't mind it

I don't live any secret life

I don't call or text very much.

I didn't mind this part of the study much.

I didn't delete anything. I hope this info helps the study.

I did, t care

I did not worry about study having access to my info.

I did not think about it.

I don't think it influenced my recovery at all but i can see how it may be helpful to some

I am only sorry that my info is not more entertaining

I am just hoping the information offers insight. I don't have privacy concerns, really.

Fine

Fine

Doesn't really effect me

Doesn't bother me

Didn't think much about this

Didn't mind

Didn't bother me.

It feels Invacive but I didn't have any issues with it

All good

Again, no. Issue

Again, i don't even think about it

Again, helpful

Again didn't think about it much at all

Accountability is important

Too personal

This is becoming a bit more personal , but seems like valuable info

Somewhat of a privacy issue.

Some things were personal and sometimes i forgot they were being looked at

Occasionally i would say something intimate on text that i didn't want to share otherwise it was fine

Not being able to clear my messages and keep my phone „!,cleaned up,! bugs me!

Never really understood what the study was going after with my log and what it meant to the survey and rexocefy

My only problem was having the text message content tracked because my girlfriend said she didn't wanna to send me nudes because she knew the study people would see it

Just in general this feels intrusive but as part of the study it is fine!

It's intrusive , but i understand the utility.

It's annoying to be tracked but if it helps

It would probably be more effective if you had a way to include Facebook messages and the like.

It seems this was more to aid with the study research than to help with my recovery. If i could see a way it would be prioritized to help me, i would be more willing to have them tracked longer. Because of the way its use was communicated to me, it didn't seem to improve accountability.

It feels like an invasion of privacy.

I wouldn't let my text message contents be tracked if i hadn't been paid

As i said in previous comment, i trust no one tracking my information. Too much of our privacy and protection is lost already In today's society. I think if someone was really bad off and could not recover from substance abuse, tracking on this way may be a good idea to help that person maybe see more what is tripping them up, like possible patterns that may lead to their failure. In that case if other things did not work, i would be more likely to opt for more phone monitoring.

I suppose for a year study I would be concerned about misuse of data.

I'm not sure here. I was never a social drinker, i.e. I usually drank at home with at most one other person, so you wouldn't see texts like hey man, let's go get drunk. i say Undecided here not because the data is useless, but I struggle to see how, at least for me, it would be useful.

I have a lot of conversations that I don't feel comfortable sharing with the staff. The call logs are not a big deal for me to be tracked but I feel as though access to text content can be intrusive.

I don't feel comfortable sharing my text messages.

I don't care for the intrusiveness of it but for the studies sake I do it.

I do not like people reading my messages

I disliked saving my text messages. I like deleting them when I'm done

I didn't like not being able to delete calls and texts. I like a tidy phone.

I did not use the texting options and preferred using the phone calling options instead.

Hate the idea of someone reading my text messages.

Generally didn't matter except for occasional work or personal texts to my partnee

Call and text logs are a little personal. Would prefer not to share them but understood it was a part of the study

At first it is uncomfortable having to share messages documenting emotional immaturity and all the effed up co dependent relationships i have with some folks, but i focus on the study goal. It is much more important to me to create tools that prevent disaster and death than for me to impress some people i don't really know. Frankly i already damaged my reputation my self with my reliance on substances and subsequent shitty behavior. I'm kind of callous in that respect. It also helps that i don't have illegal streams of income to protect. And we signed a waiver. I trust you all are professional.

Again, total invasion of privacy.

Again, it freaks me out that you can know everything I say. I sometimes say sensitive things.

Again, i did not think much about it until check in dates were approaching, and i would become a little concerned about more intimate conversations being less than private.

Again at the beginning of the month other people were using my phone and I usually have all my numbers saved that I know

Really not sure about this one

Not sure if this whole situation is bothers I'm to me yet.

Not fond of it but if it helps than it makes sense

Kind of creepy but it is harmless.

Just kind of weird but it's anonymous so didn't bug me much.

It's a pretty personal thing but I understand the reasoning behind that

I'd rather not.

I see the reason for the tracking (pattern gathering and all that groovy stuff) but I, Christopher Robin, had identified those who are my friends and those who just want to party. It was painful but the latter have been put at arms length

Also strange

Who cares

Pretty much same answer as previous.

Nuthing really

Nothing really

Nothing

Nope

None

None

No comments

No comment

Na

Na

Na

Big data

Sleep Quality

Table S5: Participant Free Response Comments about Sleep Quality ($N = 78$)

Comment
Using the sleep monitor was not an issue at all. Very simple to use and set up. I will likely purchase this monitor to continue using now that the study is complete. Every one in awhile i would have to reasjust, this was not an issue
The sleep monitor was fun to use, which helped keep me on track for sleeping. It holds you accountable for a specific bedtime in a way because you want to try to keep a consistent score. You can't feel it so it doesn't disturb sleep unless you're really tired and forget to push the sleep button.
The sleep monitor was cool. My partner and I are thinking of buying our own.
Seeing quality sleep. Time was cool
Positive experience. I liked thinking about my sleep and then checking if the monitor recorded my expectations.
I'm convinced that it doesn't report accurately. It's kinda fun though
It's cool. It's nice to be able to see how you're sleeping, especially how trends develop over time.
It wasn't inconvient at all
It wasnt bothered at all by the beddit. It was fun to use but I think I need to take more time to ensure it's in the best position because I constantly roll off of it or have it in a position that prevented measurement.
It was very interesting to watch my sleep improve overall
It was easy and fun to use. I could validate progress on mindfulness and other efforts as i worked on myself
Meme really, it is interesting to look at the stats though
It is good to know the sleep pattern and i guess, it helped me in maintaining my sleep cycle. Thanks.
I found it hard to remember every night, setting an alarm helped. It also helped give me a sense of routine when i slept at home.
I was curious each day to see results
I may buy one
I liked the insight
The heart rate curve was often incomplete. I liked reviewing my sleep patterns.
I liked reviewing my sleep patterns
I liked it. I enjoyed getting my sleep score in the morning. I liked the alarm feature. It made me want to get better rest.
I like the sleep monitor to see how will i sleep
I found it interest I found it interesting
I enjoyed using the sleep monitor to track my sleep patterns. It was helpful to receive a score each morning
Found it very informative with heart rate, etc.
Forgot a few times. User error, quite simple to use

Easy to use and not a problem,. To make it better I'd ask for a longer strip as I had a king bed and never was sure if I w number a on the strip. Also being able to run the sleep strip through the. Computer would be helpful. I'd tend to accidently shut the app off or move my phone away from the strip and loose the connection, but the computer 'was always by my bed

Easy to use

Bedding helped me so much. I knew my sleep was bad but not that bad.

Am considering buying one

Very interesting

Thumbs up

They are positive

There were no issues with the sleep monitor

T It's interesting

Overall good experience no issues

No problems

No prov

No issues with it, only had a few nights when the readings seemed real innacurate. My biggest issues were just fears that I would crinkle it irreperably while sleeping on top of it or during transport. But that never really happened.

No issues to report

Like it. Didn't bother me

It's fine. No problems whatsoever

It's a very cool piece of technology. I'd like to know how it works since I'm an engineer.

It. Ok

It was actually pretty cool

I liked it. I just forgot to turn it on once in a while

I like using it

I have a clap for sleeping

I enjoyed using it.

Didn't affect much at all.

All positive

Would have to wake up to use.

Would be better if it would just connect automaticly

Usually I would just forget to turn it on

This month was hard because of winter break. Stayed in so many different places

There were problems with the sleep monitor in that I was not able to activate the monitor when I was going to sleep. I simply could not activate it and I believe my home location had a lot to do with it.

The sleep monitor was at first intriguing but the novelty soon wore off. I really don't think it is that accurate as there were nights I slept the sleep of the dead but received an atrocious score upon waking up. Conversley, there were times I barely slept and rocked it. Meh, who knows.

The sleep monitor was annoying. I think you should advise participants to set an alarm to remind them to turn it on.

The only problem i had was sometimes it would disconnect in the middle of the night.

Sometimes it recorded all night but when I pressed I'm up there was no data recorded

Relatively innocuous device, but I travel a lot

Often loses the heartbeat.

Just irritating.

It was difficult to use the monitor as I was transitioning between multiple locations.

It kept me away from my partner at night which was not good for us.

It feels somewhat intrusive to use in that I am a rather private person in some areas of my life. I question its accuracy as there are a few times i was up and out of bed that did not register.

It didn't seem to work reliably for me, so I think that was the biggest cause of my reluctance to use it/spend time (albeit only a few seconds) adjusting the monitor strip to be properly under me

It could be difficult to remember to use.

I wish I used it with my iPad because Bluetooth killa my batteries and I use my phone as an alarm.

I take a lot of naps, which significantly interferes with its accuracy.

I liked it i wish it cover more of the bed because sometimes i rolled off of it

I just had problems with connecting some nights. Also i got a different mattress and i had problems with it reading.

I had problems using the sleep monitor.

Forgot to turn it on frequently

Fine until it called me at 4 am

Drained phone battery

Didn't work on some nights.

Nothing really

It was fun data to look at each morning. A bit of routine work to keep in place though

EMA

Table S6: Participant Free Response Comments about 4X Daily EMA ($N = 153$)

Comment
When experiencing depressive thoughts, taking the survey drew attention to those feelings. When doing the survey, it's a moment to take stock of feelings (aroused/alert vs. calm/sleepy) and successes or failures with maintaining abstinence. Definitely impacted my mood overall. Especially the audio surveys.
Very helpful and a constant reminder of why I'm moving forward
Thought they helped with focus and accountability
They're helpful and remind me that I'm trying to stay sober
They were quick and easy to complete though it could be easy to want to straight line answer on occassiob
They were fine unless I was workig, for some reason I thought I had to complete within 1 hour
They were a good way to stay thinking about recovery.
THey were a good check in tool throughout the day
They kept me present
They help stay honest with myself
They were often e The tin end The timing Ms number per The timeliness and number of surveys daily keep one in check for staying on track. The only negative part of the surveys for me was the audio,as I felt I didn't really have any thing to add. Being they were daily the repetitive response from me wasn't helpful to the study. I think the visual I refraction would be more beneficial.
The surveys were not a hassle and they helped me become more in tune with my feelings. It's nice to have an hour to complete the surveys in cases when they send at inconvenient times.
The surveys were a helpful was to keep me cognizant of my recovery efforts
The surveys trained me to check in with my self.
I felt it was an annoyance, another thing i had to do on top of everything else when it was so frequent. I found one per day is tolerable by days end not a set time. I am just too scattered with how my brain works. Also it was a reminder of having a problem with alcohol which could be good or bad depending on how I came it mentally. If i think of it as a reminder of how great i am doing daily despite my life struggles then it is good. The surveys are good for making me check in with myself. That is good! Self reflection.
The surveys are a great reminder of my sobriety
The survey was great for the first three months as i worked on what and how i was feeling and attempted to increase how mindful i was of my feelings. Fourth month i found myself trying to detach more from my phone and stay in the moment so it was harder to complete
The home surveys were easy to complete but I would have inserted different questions to vary the routine.
The daily surveys were not an inconvenience. I think they helped increase my awareness of how drinking made me feel bad the next day and raised my awareness of situations where I was likely to drink (like with my Mom and on dates) and situations that I could make better choices (like picking restaurants that didn't serve alcohol for dates)
The daily survey were good for me kept me on track

Surveys were not a problem, sometimes cumbersome, but usually welcome. And quick, easy to keep up with

Survey helped me see how my emotions changed

Somedays it would be tough to get to them, but usually provided a good moment of clarity

Simple and easy.

One daily would be great

My surveys ended a couple of days ago. I miss being reminded to check my emotional state

Makes you think more about your day and what you should be doing

The only real problem with the survey is , like AA, it relies on the participant being honest about what's happening. One could easily lie just to alleviate guilt about drinking. That said, it was made clear to me from the beginning that I wouldn't be judged or punished for failing to stay sober, so I was comfortable being honest.

It was interesting to notice my moods and connection of them to alcohpl

It was a nice daily check in on how my days were going. The surveys help gauge my current feelings instead of bypassing what I feel intangibly.

It was a good tool to help me reflect. However, when i was busy or travelling it felt a bit more like a chore. Especially during the last weeks

It was a good check in for my emotions

It provided additional accountability

It kept me accountable overall

It is a good check in

I missed not getting my text this week! It was a bit challenging in making my appointmenta with having started a new job and new clarity of being sober. It helped me to be mindful about where i would go for dinner etc. The obsession lifted about a month in. No idea but it drinking was not my first thought in the morning or last thought at night. It's kind of amazing how it works still fantasize about what certain drinks taste like and the felling i would get from one drink. But i would quickly keep the tape running to where my day would go so quickly out of control. I want to thank all of you for being part of my recovery story.

Good. It helped me keep me honest by reminding me that i can't have alcohol. Four reminders per day to abstain.

It did help in keeping recovery a focus in my life

I think the daily secrets were helpful with my recovery because they kept the issue in my forethoughts

I think knowing i had the surveys to answer positively reinforced my decision to not drink

I still don't really have strong feelings one way or the other. I suppose the surveys popping up at random times is kind of a good way to trigger a little self-analysis throughout the day, which is generally think is not a bad thing.

I liked them because they helped me take note of my emotional health

I liked the structure that it added to my day. For other activities,a being deliberate about setting aside time for things has helped me maintain changes to my life for the better. The surveys were no exception.

I like the surveys because I was able to check in with myself and how i was feeling.

I like the focus for me

I like how the surveys give me a time to reflect on how my day is going. I was just thinking earlier today how I'm actually going to miss them and feel weird not taking them every day. I wouldn't recommend doing more than 3 in a day though.

I had no issues for the daily surveys. I felt that it kept me in check and reminder to not drink. I would not change it.

I found them encouraging and i also thought they were helpful in the sense that they made me 'stop and think' about how i was feeling, cravings, etc.

I found the surveys helpful, good accountability

I enjoyed talking the surveys. They very interesting.

I k I enjoyed it. It reminded me that i was in recovery, what i would lose with relapse, and haA a calming effect.

I enjoy checking in it keeps me thinking throughout the day

I didn't mind doing the surveys, as long as I had Internet there were no burdens related to getting them completed. Sometimes I would miss them for various reasons, which was a little anxiety provoking at first when I was very worried about missing them, but after time this was no longer too large of a concern, as I became more comfortable.

I believe the study was a big part of my recovery and one of the biggest reasons I'm still sober today.

I believe the program helped keep me accountable and kept abstaining on my mind

Helps to keep me acountable

Helped me check in with myself

Helped keep me accountable and keep me want to stay sober

Great tool for self check in. Not necessary to do four per day

Good, helped me not drink.

Generally i miss the emotional check in and level of urge to drink. It helped me stay mindful throughout the day.

Easy and straight forward

Easy and simple. Not problems with them. I would like to see on the text a. Reminder what the cut off time for getting them done was

Staff alone with the student reacher. Thank you this during this time i was able to game further insite to my life chooses.

Didn't bother me. Kept me in check, which was positive for my recovery

Daily surveys reminded me of my goals daily

At times it was inconvenient but if it helps with recovery its worth it

Allows me to reflect and focus

They were fine just sometimes bad timing

They were fine, but if I missed one, I wasn't sure if I should do both and was worried I'd do them in the wrong order.

They didn't interfere, but sometimes they would be sent at times when I was busy

They are good

They all started to blend together

Surveys were great. I had gotten so used to them coming that i miss them now that they are no longer coming!!

Surveys were fine hi Surveys were fine only thing i didn't like was the first one of day and having to predict if i was going to drink that Week or not. Would've helped if it was only for that day. That would've been an obtainable goal that i could focus on

Relatively Unobtrusive

Positive I thought it was a good thing

Positive

Overall good experience but did have issues receiving them sometimes

Overall a nice experience

Noticed the surveys appearing when near certain facilities.

No problems

No problem sometimes I don't carry my phone miss the survey

No issues

My answers were always the same, but i guess that is good

It wasn't a hassle for me at all.

It was fine with me I'm just ready to move on.

I would be willing to extend the survey

I love this program

I liked these

I just like doing them

I got used to them. It was strange not to do them. It might help with accountability.

I got used to doing the surveys. Expected the notifications at 37 past the hour!

I found them helpful

I didnt mind the surveys but after the second month i felt like they became repetitive and I didn't feel like I got much utility from them

I didn't mind the process too much, but as time went on, I payed less attention to giving thoughtful answers each time.

Has been positive for me

Great

All good

Well they haven't been functioning properly for me as you know. So, I didn't like the in predictability of them. I also don't like how it seems like when you say have had a drink since the last survey it gives you the little calendar thing and a drop down menu to select the time, but it's only in one hour increments and also its not clear how to indicate that you're still drinking at the moment you're filling it out.

They were a hassle

They often made me think about alcohol.

They could have come more spread out throughout the day. The second one usually came within an hour of the first one. The third was usually during work hours as well.

There were times it did interfere with daily activities or i wud get a reminder even and still forget to do it, but the reminders for the daily survey helped a lot

There were some daysbibfelt like just walking away from my phone so it was hard to keep checking it in order to do the surveys

The surveys needs to evolve or be adjusted to my needs as an addict in treatment. The questions repettwisty nature makes me feel as if my recovery is stuck in neutral. The questions the user needs to respond to on, say day 65 of the recovery, the user feeljng

The inconsistent times of receipt is what mostly posed the inconvience, in addition to potential skewing collected data and /or result. Not only would I sometimes receive 3 or was prompted to complete more than 4, the times throughout the day were never the same. For eg, I would receive the 1st, longer survey typically at 8am, and then the 2nd follow-up right away at 9 and other times at noon. If I were sleeping in, I would answer the exact same for both, which I feel was unnecessary and unhelpful towards the research. I think it would be more beneficial in gen. To both the participant and researcher to have only 3 surveys (vs. 4) sent out in the morning, midday, and evening.

The four surveys were too much

The four interruptions are rather a pain. I have wanted to yell -well NO i had not thought about drinking but now that YOU mentioned it I want a drink!!-

Surveys a bit intrusive. One or two less surveys a day would be ideal.

Sometimes i forgot about the audio survey, and sometimes was busy for the written.

Sometime. It. Got on my last.

Seemed like a lot when in was busy with kids and family stuff. I probably answered out of habit more than what was true in that moment

Once i began drinking i had an aversion to the surveys

On the question How are you feeling right now?. The only options are Aroused and awake or Calm and sleepy. But sometimes I feel sleepy but not calm and sometimes I'm calm and awake so it was difficult to rate myself.

My state cannot be measured so simply, for example I am often aroused (don't mean sexually) and tired at the same time, but they are at opposite ends of your spectrum.

Just very repetitive n ofter

Just too many.

It's just annoying if I'm out hiking all day or if I'm out at night because I don't like to be on my phone at all in those situations.

It's difficult to predict possible stressful or risky situations for the future.

It was difficult when at work.

Interfered with work

If I'm busy hard to complete all the surveys

I wasn't always good at completing the surveys. My phone in general can be a bit of a stressor, so there were times I just wanted to keep my phone off.

I just starting getting tired of them. If the questions had a little more variety it might have helped but I understand they needed to be consistent for the purposes of the study.

I just sometimes would miss them throughout the day because i would have to silence my phone

I found 'at risk situations or people' did'nt always match the answers I gave, depending on how confident I felt at the time. I came to sometimes enjoy recording my audio survey. Maybe someone was actually listening to me!

I don't really see much of a correlation with completing these surveys and staying sober

I did well with my recovery on my own and the surveys were more of a burden at the end. Maybe base the number of surveys on each individual's recovery progress.
Hard to stop what your doing at work to be on your phone. Does not look good at work.
Four surveys were a bit much.
Finally did start to bug me when they popped up
Didn't like doing them once i went back to work.
Completing the surveys four times a day did become boring and repetitive some days. I didn't feel like my mood or thoughts changed much over the course of a single day
Became overwhelming over time
At times it was difficult to complete every survey as i was at work, busy with the kids, etc. I found myself feeling guilty when i missed and this caused some stress.
At times inconvenient
As I got busier it was harder to keep up with the surveys
With my insomnia and untreated adhd I sometimes forgot or ended up doing them late and my sleep schedule is sparatic
They are redundant. When I had the flu. Should be included question on illness.
Sometimes it's annoying
Minor inconvenience
It could come up at inopportune times.
I understand why I do them but it gets a bit tedious.
I miss some surveys due to my new job. We can't have our phones on us
Gets a bit old after a while
At the end, a Ginal Thanks for participating would be nice
Nothing really
None
No thoughts
Na
It was a dog grant experance
I kept forgetting to do the voice messages. I would get side tracked or forget what to say.

Sample participant free-responses mapped onto each theme

Table S7 presents 5 themes that emerged in participants' free-response feedback about each personal sensing data stream: acceptability, sustainability, benefits, trust, and usability.

Table S7: Sample Participant Comments

Theme	Sample Comments
**Acceptability**	"I had no issues for the daily [EMA] surveys. I felt that it kept me in check and were a reminder to not drink. I would not change it."

	"I liked the structure that it added to my day. For other activities, being deliberate about setting aside time for things has helped me maintain change to my life for the better. The [EMA] surveys were no exception."<br>"They [EMAs] were quick and easy to complete."<br>"I felt it was an annoyance. Another thing I had to do on top of everything else when it was so frequent. I found one [EMA] per day is tolerable. I am just too scattered with how my brain works."<br>"The [EMA] surveys need to evolve or be adjusted to my needs as an addict in treatment. The questions' repetitive nature makes me feel as if my recovery is stuck in neutral."<br>"Great tool for self check in. Not necessary to do four [EMAs] per day."<br>"It takes time out of your day where you have to completely switch locations just so you can do it [audio check-in] in private. I don't like that people could hear me and the topic wherever and whenever so I stopped using it."<br>"I also have to keep privacy in mind since it [audio check-in] is not something I'd like to complete within earshot of others."<br>"The only negative part about the daily [audio] check in was my mom, who I live and work with, listening in while I was talking. I would have to find a time when she was preoccupied or away from me to talk freely."<br>"I loved this part! It was like journaling kind of where I would discover things that were hidden in my subconscious. I have a recording app on my phone and I will continue doing the audio check-in as a form of checking in with myself."<br>"This [audio check-in] was my favorite part of the study. It helped me to set a good intention towards my recovery."<br>"I liked the daily [audio] check in. It gave me a way to vent without being judged."<br>"Location tracking is fun and useful for me personally in addition to the study."<br>"It was interesting to see my [geolocation] patterns and places."<br>"No issues. I like that they allow me to delete some items from the [cellular communication] log if I wanted to."<br>"Having the opportunity to delete texts was nice even though I hardly ever did."<br>"I have no issues at all with tracking my calls."<br>"Call and text logs are a little personal. Would prefer not to share them but understood it was a part of the study."<br>"Positive experience. I liked thinking about my sleep and then checking if the monitor recorded my expectations."<br>"I enjoyed using the sleep monitor to track my sleep patterns. It was helpful to receive a score each morning."<br>"The sleep monitor was annoying. I think you should advise participants to set an alarm to remind them to turn it on."
**Sustainability**	"I forgot I was being tracked [geolocation] so it was not a big deal to me."<br>"I forgot this was even happening most of the time. It [geolocation] did not interfere with my life."<br>"I went about my day as I normally would and never thought about it [cellular communication logs]."<br>"Never noticed so it [cellular communication logs] was zero inconvenience whatsoever!"<br>"Would agree to extend [cellular communication logs] to a year no problem."<br>"My [EMA] surveys ended a couple of days ago. I miss being reminded to check my emotional state."<br>"I didn't mind the [EMA] surveys but after the second month I felt like they became repetitive and I didn't feel like I got much utility from them."<br>"[4X EMA] became overwhelming over time."<br>"One daily [EMA] would be great."<br>"I did well with my recovery on my own and the [EMA] surveys were more of a burden at the end."<br>"The daily [audio] check-in become more difficult to complete as time went on."

	"The sleep monitor was cool. My partner and I are thinking of buying our own."
**Benefits**	"I felt all right having my location tracked. If it were used in a way to keep me from relapsing my feeling about it would be even more positive."
	"I'm a somewhat typical introverted, slightly paranoid, grump, so the idea of [geolocation] being tracked is automatically negative. That being said, if it's something that helps people that can be a really good thing."
	"It seems this was more to aid with the study research than to help with my recovery. If I could see a way it would be prioritized to help me, I would be more willing to have [cellular communication logs] tracked longer."
	"Was okay to have [geolocation tracking] done in the context of the study or for an app that would help me stay sober."
	"If it [cellular communication logs] helps recovery, fine with me!"
	"It [cellular communication logs] is a huge invasion but my sobriety is paramount to my overall health."
	"Loss of privacy for the sake of sobriety, okay I'll give in [cellular communication logs]."
	"If it [geolocation] helps me then I would do it."
	"I wouldn't let my text message contents be tracked if I hadn't been paid."
**Trust**	"I trusted the study group to not use my personal information for any other use."
	"I don't mind turning this info [cellular communication logs] over and I trust the people running the study."
	"I don't mind this at all, as long as I know this information [cellular communication logs] is protected and anonymous."
	"Didn't bother me since it [cellular communication logs] is protected."
	"Just in general this [cellular communication logs] feels intrusive but as part of the study it is fine!"
	"I am concerned with data privacy, and felt uncomfortable having my location actively tracked."
	"I suppose for a year study I would be concerned about misuse of [cellular communication log] data."
	"I have a lot of [text message] conversations that I don't feel comfortable sharing with the staff."
**Usability**	"I experienced issues with this [audio check-in] from the very beginning and discontinued early on. I was unable to get the message beyond 8-10 seconds with close to 6 attempts each day. Was not worth the time or stress for me."
	"My bigger problem with the [geolocation] tracker app was more the fact that my battery power was getting depleted so rapidly, sometimes within 2 hours post-full charge. I found myself charging my phone at least 5 times a day. And at times, this became very stressful."
	"There were problems with the sleep monitor in that I was not able to activate the monitor when I was going to sleep."
	"Would be better if it [sleep monitor] would just connect automatically."
	"It was difficult to use the [sleep] monitor as I was transitioning between multiple locations."
	"Not being able to clear my [text] messages and keep my phone [log] cleaned up bugs me!"
	"I disliked saving my text messages. I like deleting them when I'm done."
	"I didn't like not being able to delete calls and texts. I like a tidy phone."

Note:
Green text indicates positive comments about sensing method. Red text indicates negative comments and black text indicates neutral or mixed comments.

Group differences between continued and discontinued participants

We ran a series of t-tests to compare demographic information and information relevant to participants' alcohol use in participants who continued vs. discontinued. Continued participants included all participants who were used in the study analyses (i.e., completed through first month followup; $N = 154$). Discontinued participants consisted of eligible participants who did not enroll or discontinued prior to the first month follow-up ($N = 36$). We found no significant differences between groups (Table S8). Note that we are missing demographic data for 1 participant who consented but did not subsequently enroll in the study.

Table S8: Pairwise T-tests to Compare Group Differences between Continued ($N = 154$) and Discontinued ($N = 36$) Participants

	t	*df*	*P*
Hispanic	-0.06	51.07	0.95
age	1.06	48.79	0.30
avg_age_aud_milestones	0.94	56.38	0.35
dsm5_total	-0.96	57.66	0.34
education	1.70	46.30	0.10
employed	0.35	51.60	0.73
income	0.93	52.83	0.36
male	-1.21	53.17	0.23
married	0.67	56.12	0.51
num_other_drugs	0.59	55.14	0.56
num_quit_attempts	-0.75	84.11	0.46
num_types_tx	0.52	53.36	0.61
white	-0.31	54.86	0.75

Reasons given for participant discontinuation

We provide a more granular breakdown of reasons for participant discontinuation in Table S9 below.

Table S9: Characterization of Discontinued Participants

	n	%
Eligible and consented participants discontinued prior to completing enrollment		
Health concerns	1	4.5
No longer has transportation	1	4.5
**No longer interested**	**3**	**13.6**
Participant rescheduled multiple times before cancelling/no showing	7	31.8
Unknown	10	45.5
Total	22	100.0
Enrolled participants discontinued prior to first month follow-up		
Family crisis	1	6.7
**No longer interested**	**1**	**6.7**
No longer sober or no longer wishes to abstain from alcohol	5	33.3
**Noncompliance with providing data**	**1**	**6.7**
Participant rescheduled multiple times before cancelling/no showing	3	20.0
Unknown	4	26.7
Total	15	100.0
Enrolled participants discontinued after the first month follow-up		
Cell service shut off	3	14.3
**Cited study demands as too burdensome**	**1**	**4.8**
Moved out of state	2	9.5
**No longer interested**	**3**	**14.3**
No longer sober or no longer wishes to abstain from alcohol	3	14.3
**Noncompliance with providing data**	**2**	**9.5**
Participant rescheduled multiple times before cancelling/no showing	4	19.0
Unknown	3	14.3
Total	21	100.0

Note:
Bolded rows depict explicit acceptability-related discontinuation, but no discontinuation can be definitively established as unrelated to acceptability.
