# Supplementary material for: Acceptability of Personal Sensing Among People With Alcohol Use Disorder: Observational Study
Source: JMIR Mhealth Uhealth. 2023 Aug 28;11:e41833. doi: 10.2196/41833 (PMC10495858; doi:10.2196/41833)
Supplement: Multimedia Appendix 3 [file mhealth_v11i1e41833_app3.docx]

# Acceptability Survey

These questions ask you about your experiences with the wristband, phone, and other technology in this study **during the last month**.

Please indicate how much you agree or disagree with each statement about your experience **wearing the wristband during the last month.**

1. The wristband interfered with my daily activities.

Strongly disagree Disagree Undecided Agree Strongly Agree

1. I disliked wearing the wristband.

Strongly disagree Disagree Undecided Agree Strongly Agree

1. I would be willing to wear the wristband for one year if it helped with my recovery.

Strongly disagree Disagree Undecided Agree Strongly Agree

1. Tell us your general thoughts, whether positive or negative, about your experience wearing the wristband.

______

Please indicate how much you agree or disagree with each statement about your experience **using the sleep monitor during the last month.**

1. The sleep monitor interfered with my sleep.

Strongly disagree Disagree Undecided Agree Strongly Agree

1. I disliked using the sleep monitor.

Strongly disagree Disagree Undecided Agree Strongly Agree

1. I would be willing to use the sleep monitor for one year if it helped with my recovery.

Strongly disagree Disagree Undecided Agree Strongly Agree

1. Tell us your general thoughts, whether positive or negative, about your experience using the sleep monitor.

______

Please indicate how much you agree or disagree with each statement about your experience **completing the daily surveys during the last month.**

1. Completing the daily surveys interfered with my daily activities.

Strongly disagree Disagree Undecided Agree Strongly Agree

1. I disliked completing the daily surveys.

Strongly disagree Disagree Undecided Agree Strongly Agree

1. I would be willing to complete four daily surveys a day for one year if it helped with my recovery.

Strongly disagree Disagree Undecided Agree Strongly Agree

1. I would be willing to complete one daily survey a day for one year if it helped with my recovery.

Strongly disagree Disagree Undecided Agree Strongly Agree

1. Tell us your general thoughts, whether positive or negative, about your experience completing the daily surveys.

______

Please indicate how much you agree or disagree with each statement about your experience **responding to the daily recovery check-in during the last month.**

1. Responding to the daily recovery check-in interfered with my daily activities.

Strongly disagree Disagree Undecided Agree Strongly Agree

1. I disliked responding to the daily recovery check-in.

Strongly disagree Disagree Undecided Agree Strongly Agree

1. I would be willing to respond to a daily recovery check-in for one year if it helped with my recovery.

Strongly disagree Disagree Undecided Agree Strongly Agree

1. Tell us your general thoughts, whether positive or negative, about your experience responding to the daily recovery check-in.

______

Please indicate how much you agree or disagree with each statement about your experience **carrying the iPhone everywhere during the last month.**

1. Carrying the iPhone everywhere interfered with my daily activities.

Strongly disagree Disagree Undecided Agree Strongly Agree

1. I disliked carrying the iPhone everywhere.

Strongly disagree Disagree Undecided Agree Strongly Agree

1. I would be willing to carry the iPhone everywhere for one year if it helped with my recovery.

Strongly disagree Disagree Undecided Agree Strongly Agree

1. Tell us your general thoughts, whether positive or negative, about your experience carrying the iPhone everywhere.

______

Please indicate how much you agree or disagree with each statement about your experience with the **iPhone saving information about your location during the last month.**

1. I disliked having my location tracked by study staff.

Strongly disagree Disagree Undecided Agree Strongly Agree

1. I would be willing to have my location tracked by study staff for one year if it helped with my recovery.

Strongly disagree Disagree Undecided Agree Strongly Agree

1. Tell us your general thoughts, whether positive or negative, about your experience with the iPhone saving information about your location.

______

Please indicate how much you agree or disagree with each statement about your experience with the **iPhone saving information about your text messages and phone calls during the last month.**

1. I disliked having my text message logs (i.e., dates, times, and phone number of contact for text messages) tracked by study staff.

Strongly disagree Disagree Undecided Agree Strongly Agree

1. I would be willing to have my text message logs (i.e., dates, times, and phone number of contact for text messages) tracked by study staff for one year if it helped with my recovery.

Strongly disagree Disagree Undecided Agree Strongly Agree

1. I disliked having my text message content (i.e., the actual text message information itself) tracked by study staff.

Strongly disagree Disagree Undecided Agree Strongly Agree

1. I would be willing to have my text message content (i.e., the actual text message information itself) tracked by study staff for one year if it helped with my recovery.

Strongly disagree Disagree Undecided Agree Strongly Agree

1. I disliked having my call logs (i.e., dates, times, and phone number of contact for phone calls) tracked by study staff.

Strongly disagree Disagree Undecided Agree Strongly Agree

1. I would be willing to have my call logs (i.e., dates, times, and phone number of contact for phone calls) tracked by study staff for one year if it helped with my recovery.

Strongly disagree Disagree Undecided Agree Strongly Agree

1. Tell us your general thoughts, whether positive or negative, about your experience with the iPhone saving information about your text messages and phone calls.

______
